# What to Expect (and What Not) from Dual-Energy CT Imaging Now and in the Future?

**DOI:** 10.3390/jimaging10070154

**Published:** 2024-06-26

**Authors:** Roberto García-Figueiras, Laura Oleaga, Jordi Broncano, Gonzalo Tardáguila, Gabriel Fernández-Pérez, Eliseo Vañó, Eloísa Santos-Armentia, Ramiro Méndez, Antonio Luna, Sandra Baleato-González

**Affiliations:** 1 Department of Radiology, Hospital Clínico Universitario de Santiago, Choupana, 15706 Santiago de Compostela, Spain; 2 Department of Radiology, Hospital Clinic, C. de Villarroel, 170, 08036 Barcelona, Spain; 3 HT Médica, 14012 Córdoba, Spain; 4 Department of Radiology, Hospital Ribera Povisa, Rúa de Salamanca, 5, Vigo, 36211 Pontevedra, Spain; 5 Grupo Recoletas, 47007 Valladolid, Spain; 6 Department of Radiology, Hospital Universitario Nuestra Señora, del Rosario, C. del Príncipe de Vergara, 53, 28006 Madrid, Spain; 7 Department of Radiology, Hospital Universitario Clínico San Carlos, Calle del Prof Martín Lagos, 28040 Madrid, Spain

**Keywords:** computed tomography, dual-energy CT, iodine map, material

## Abstract

Dual-energy CT (DECT) imaging has broadened the potential of CT imaging by offering multiple postprocessing datasets with a single acquisition at more than one energy level. DECT shows profound capabilities to improve diagnosis based on its superior material differentiation and its quantitative value. However, the potential of dual-energy imaging remains relatively untapped, possibly due to its intricate workflow and the intrinsic technical limitations of DECT. Knowing the clinical advantages of dual-energy imaging and recognizing its limitations and pitfalls is necessary for an appropriate clinical use. The aims of this paper are to review the physical and technical bases of DECT acquisition and analysis, to discuss the advantages and limitations of DECT in different clinical scenarios, to review the technical constraints in material labeling and quantification, and to evaluate the cutting-edge applications of DECT imaging, including artificial intelligence, qualitative and quantitative imaging biomarkers, and DECT-derived radiomics and radiogenomics.

## 1. Introduction

Computed tomography (CT) ranks as one of the top five medical developments in the last 50 years. CT imaging has recently experienced remarkable growth with advancements in spatial and temporal resolution, radiation dose reduction, artificial intelligence (AI) integration, and new clinical applications. As a result, CT scans will continue to be a cornerstone of modern medical diagnostics and personalized medicine. However, conventional (single-energy) CT has inherent limitations in soft tissue differentiation. Therefore, dual-energy CT (DECT) imaging (a subset of spectral CT) tries to overcome these limitations by acquiring data at two energy levels (tube voltages), allowing the differentiation of materials using the energy dependence of X-ray attenuation in any material [1,2,3,4,5,6,7,8]. DECT has been considered as the next phase of CT technology development and has emerged as a useful tool, with many clinical applications that have evolved over time. DECT shows profound capabilities for improving diagnosis based on its superior material differentiation, but also shows technical constraints that must be considered for proper use in daily practice [8,9,10]. The aims of this paper are to review the physical and technical bases of DECT acquisition and analysis, to discuss the advantages and limitations of DECT in different clinical scenarios, to review the technical constraints in material labeling and quantification, and to evaluate the cutting-edge applications of DECT imaging, including artificial intelligence, qualitative and quantitative imaging biomarkers, and DECT-derived radiomics and radiogenomics.

## 2. How DECT Imaging Works

**Figure 1:**
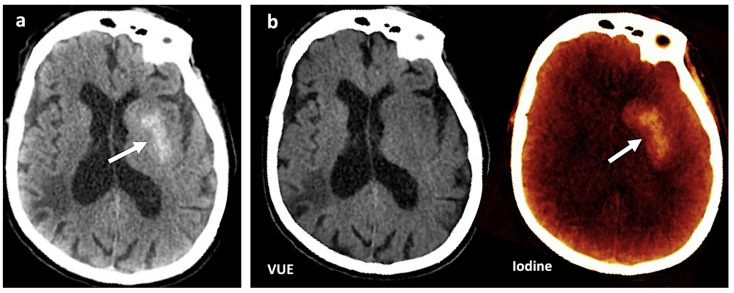
Contrast media extravasation can mimic hemorrhage after endovascular thrombectomy. (**a**) A 120 kVp-like brain image (similar to conventional single-energy imaging) shows a hyperdense area (white arrow) in the left basal ganglia following thrombectomy in a patient with ischemic stroke. Hemorrhagic transformation must be ruled out (**b**). Virtual non-contrast (VUE) and color-coded iodine overlay map evidence that this hyperdense area is not depicted on VUE while it remains on the iodine map (white arrow), representing contrast staining.

Conventional CT imaging systems use an X-ray beam that includes a wide range of photon energies (polychromatic or polyenergetic) to record the radiation attenuated by the different densities of tissues, expressed in terms of Hounsfield units (HU). CT systems measure the linear absorption coefficients of different tissues an X-ray beam passes through. The linear absorption coefficient is a result of the combination of two physical interactions of X-ray photons in the matter: photoelectric absorption (which is predominant under low energy and strongly depends on Z) and Compton scattering (CS) (which is predominant under high energy and depends on the electron density [ρ] of the material). However, single-energy CT systems show an inherent limitation in soft tissue differentiation. In conventional CT, the HU value (or CT number) of a voxel entirely depends on the linear attenuation coefficient (μ), a parameter that describes the fraction of attenuated incident photons in a monoenergetic beam per unit thickness of a material, which has considerable overlap between different body materials. If two different materials show similar coefficients (hemorrhage and iodine, for example), the same HU value will be assigned to both materials, and differentiating them will be difficult [1,5,6,7,8] (Figure 1).

**Figure 2:**
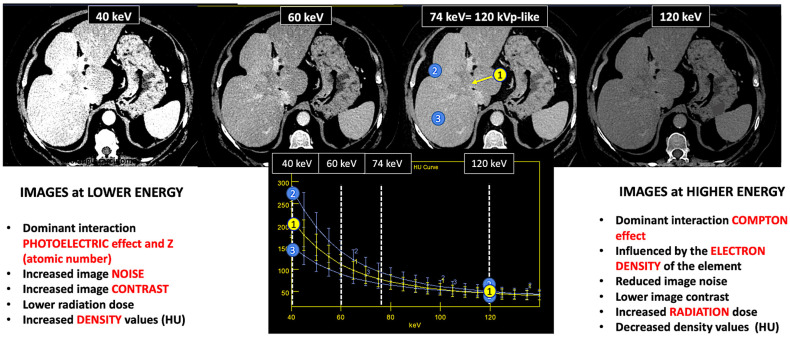
DECT images corresponding to a patient with cholangiocarcinoma evaluated at 40, 60, and 74 keV. Energy level deeply influences many image features (contrast, density values, image noise, etc.). Materials with a high atomic number, such as iodine, are susceptible to the photoelectric effect at lower energy levels. Note the differences in the spectral curves of cholangiocarcinoma (region of interest [ROI] corresponding to yellow circle 1), peritumoral area with increased enhancement (ROI blue circle 2), and normal liver parenchyma (ROI blue circle 3) due to their different iodine uptake. Furthermore, it is shown that at high energy levels (e.g., 120 keV) it is not possible to distinguish between them. Density values also change. Cholangiocarcinoma attenuation values at 120, 74, and 40 keV are 52 HU, 80 HU, and 213 HU, respectively, with increased values at lower energy.

On the contrary, DECT uses two different X-ray spectra (tube voltages) to acquire two image datasets of the same region, allowing the analysis of energy-dependent changes in the attenuation of different materials. The degree that a material will attenuate the X-ray beam is dependent on tissue composition (μ increases with increasing atomic number and increasing physical density of the absorbing material) and photon energy level (increasing with lower photon energy). Each type of material demonstrates a relatively specific change in attenuation between images obtained with a high-energy spectrum and those obtained with a low-energy spectrum. The influence of the effective atomic number (Zeff) on the attenuation (HU) at different energy values is fundamental for reliable material characterization and quantification. Although CS is the dominant interaction in CT imaging, CS does not depend on the photon energy level in the range of photons’ energies used in clinical CT. The photoelectric effect (PE) predominates at low energy values and strongly depends on the Z value of the material as well as on the energy (E) of the photons (probability of photoelectric absorption = Z^3^/E^3^). PE is the fundamental basis of DECT imaging (Figure 2).

**Figure 3:**
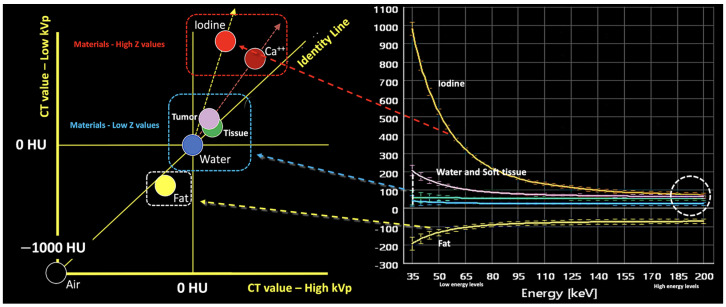
Energy-dependent X-ray absorption behavior of different materials represented as HU values using low (*y*-axis) and high (*x*-axis) energy X-ray spectra. By consensus, water and air densities are 0 and −1000 HU, respectively, at all kVp, and thus lie on the identity line. Iodine, bone, and calcium (red dotted arrow and box) demonstrate higher HU values at lower energy, with measured attenuation increasing as their concentrations increase, along lines having a characteristic slope, i.e., the “dual energy ratio” (the ratio of the CT number, in Hounsfield units [HU], of the material at low energy to the CT number of the same material at high energy). On the contrary, lighter atoms, such as most atoms in soft tissues and water (blue dotted line and box), do not present much of a photoelectric effect and show limited changes in density at low energy values. Fat and uric acid fall below the identity line, as they demonstrate lower attenuation at lower X-ray energy (yellow dotted arrow and box). Finally, note that differences in attenuation between materials are scarce at higher energies (white dotted circle, bottom right).

**Figure 4:**
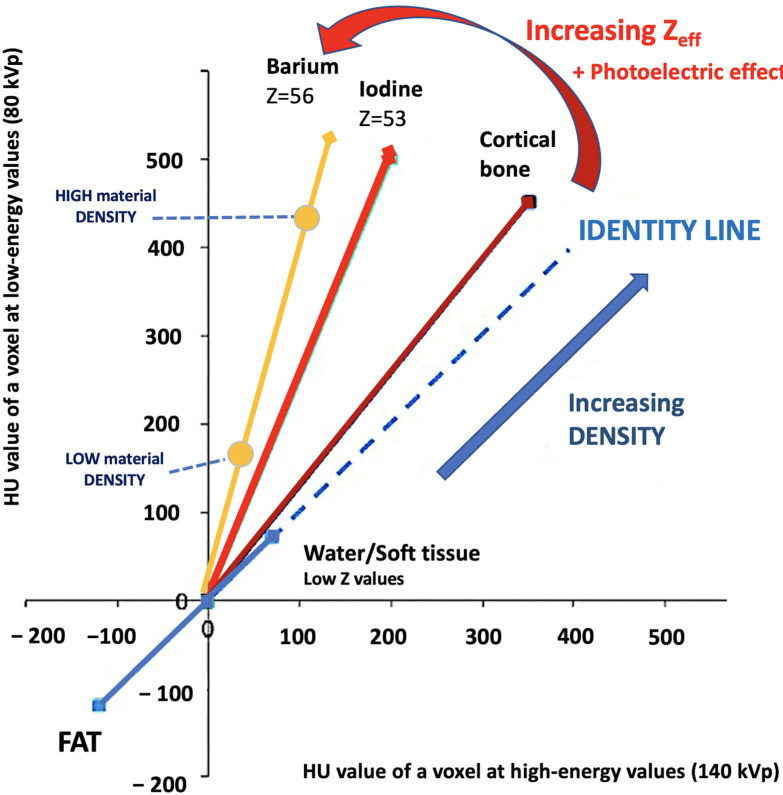
Comparing the HU value of a voxel at high (140 kVp) and low energy values (80 kVp). The slope is a characteristic of the material, and the location of the value of a given pixel along this slope depends on density. The higher the Zeff value (iodine, calcium, barium), the steeper the slope (marked increase in HU values at low energy values). On the contrary, the CT number of water and soft tissues (which have comparable Zeff values) is not energy dependent. Thus, CT numbers of soft tissues will remain almost constant when varying the X-ray beam energy.

Materials with a high atomic number, such as iodine, calcium, barium, and gadolinium, are susceptible to the PE at lower energy levels, which can be exploited to differentiate those materials. On the contrary, lighter atoms, such as most atoms in soft tissues and water, do not present much of a PE in the range of energies used in clinical CT scans. Based on the relatively specific change in attenuation with two different energies, material composition information can be obtained to allow tissue characterization [1,2,3,4,5,6,7,8,9]. In this setting, two materials may have similar attenuation coefficients in the single-energy spectrum, but may be differentiated in DECT based on their attenuation properties at two different energies (Figure 1). When CT numbers of a material at low (70–80 kVp) and high (135–140 kVp) energies are plotted along y- and x-axes, the slope is a characteristic of the material, and the location of the value of a given pixel along this slope depends on density. The higher the Zeff value (iodine, calcium, barium), the steeper the slope (marked increase in HU values at low energy values). On the contrary, CT numbers of water and soft tissues (which have similar lower Zeff values) are not energy dependent and will change very little when varying the X-ray beam energy. Therefore, these elements will remain close to the identity line (or line of equality) that represents values at which both CT numbers are same. As a result, DECT may differentiate materials if their atomic numbers differ sufficiently. Furthermore, by consensus, water and air densities are 0 and −1000 HU, respectively, at all kVp, and thus lie on the identity line [9,10,11] (Figure 3 and Figure 4).

Finally, it must be considered that spectral separation is a fundamental feature for improving DECT material labeling. Spectral separation depends on the amount of overlap between the low- and high-energy X-ray spectrum. Theoretically, it would be advantageous to use two energy levels as far apart as possible. However, there are some limitations to the energy levels that can be used in DECT imaging. So, at peak energies less than 80 kVp, too few photons are generated and a substantial proportion of them would be absorbed by the body, increasing image noise and reducing their utility in imaging. Conversely, voltages higher than 140–150 kVp are typically not available in all DECT scanners and result in higher dose of radiation with too little soft tissue contrast [11,12].

### 2.1. How DECT Characterizes and Quantifies Materials

Material decomposition using DECT imaging considers the density (HU) of a voxel with an unknown composition as a result of a linear combination of two or three (in advanced models) materials based on the change in attenuation between the two energy levels. Material decomposition algorithms can be applied to raw data (two-material decomposition) or to the image-space domain (three-material decomposition).

**Figure 5:**
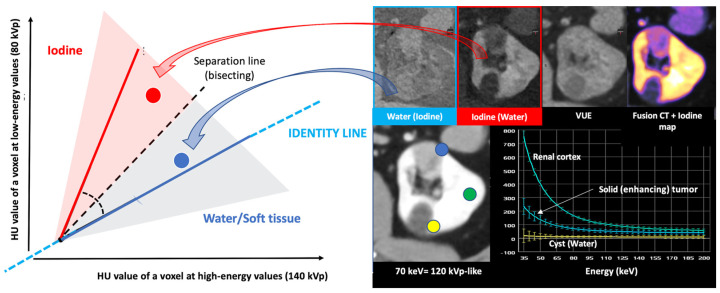
Two-material decomposition algorithms assume that the entire voxel is composed of only two preselected materials in different proportions and mathematically transform material attenuation information into the amount (or concentration) of two-material pairs that would be necessary to produce the measured attenuation level within each image voxel based on the difference in atomic numbers of the materials present within the voxel. The two-material decomposition algorithm creates material-specific image pairs. Any two materials can be selected for two-material DECT decomposition, but water and iodine are the basis pair typically used in clinical practice. Proper identification of the materials relevant to each application is paramount for accurate characterization, quantification, and subtraction (e.g., virtual unenhanced [VUE] imaging). Note the differences in a patient with a solid renal tumor (blue circle and spectral circle) and a simple cyst (yellow circle and spectral curve) comparing to normal renal parenchyma (green circle and spectral curve) in water (no iodine), iodine (no water) maps, color-coded iodine maps, and spectral curves that depend on iodine uptake.

In material differentiation or labeling, two materials with different dual-energy slopes caused by different PE effects can be differentiated. Materials with high Z values (such as iodine, calcium, or barium) can be adequately differentiated from usual body materials (hydrogen, carbon, nitrogen, and oxygen), which present a weak PE at low energy values. This is fundamental to material decomposition using DECT. The characteristics of several basic materials like iodine, water, calcium, and fat at different energy levels are well known and used for analysis. Therefore, it is possible to characterize an unknown material based on its attenuation plot at different energy levels and according to its position in relation to these basic materials [5,6,7,8,9,10,11,12,13]. Decomposition algorithms create material-specific image pairs. Any two materials can be selected for two-material DECT decomposition, with iodine–water being the most useful (Figure 5). Nevertheless, the approach of basis material decomposition differs slightly, depending on the CT system used to acquire the dual-energy data.

### 2.2. More Image Types Are Available with DECT Than with Single-Energy CT

The main advantage of DECT is the capability of offering material- and energy-selective images. The former include material decomposition with material labeling, quantification (distribution maps), and subtraction (iodine, calcium, etc.), while the latter include monoenergetic imaging, spectral curves, Zeff, and effective electron density (Rho-Z maps) [5,11,14].

#### 2.2.1. Material—Selective Images

A given basis material can be detected, labeled, and subtracted using DECT. Iodine, calcium, monosodium urate, or fat are frequently used as basis materials in clinical practice [11,14,15] (Table 1).

##### Material—Labeling

In material labeling, two materials with different dual-energy slopes caused by their PE effects can be differentiated using a pre-defined separation line. Basis material images selectively display the material in question in gray scale or with a color overlay. The most common images used are iodine maps, which specifically show iodine distribution in tissues with improved differentiation between enhanced and non-enhanced lesions. However, many other pairs of basis materials can be analyzed based on DECT, including uric acid/calcium (differentiation of uric acid from calcium in kidney stones and the monosodium urate crystals in the diagnosis and follow-up of gout patients), calcium/water or soft tissue, calcium/hemorrhage, and silicone/soft tissue [2,3,15,16,17,18,19]. Furthermore, multi-material decomposition algorithms allow quantification of the percentage of fat in a volume of tissue. Fat quantification based on DECT has been used for measuring liver steatosis, fatty bone marrow content, or myosteatosis; for evaluating adipose tissue distribution; and for characterization of adrenal gland lesions [15,16,20] (Table 1).

##### Material—Subtraction

Iodine may be subtracted from material-specific images, generating virtual unenhanced (VUE) images. VUE images have shown image quality comparable to that of true non-contrast (TUE) images, potentially obviating the need for a TUE acquisition and therefore reducing radiation exposure and scan time [21].

Virtual non-calcium (VNCa) imaging is able to estimate the amount of calcium on a DECT dataset and to subtract bone mineral or calcifications from images. Main VNCa clinical applications are the removal of calcified plaques from vessels without subtraction and the depiction of alterations on the bone marrow and cancellous bone, including bone tumors or bone marrow edema (BME) [19,22]. VNCa suppress the high attenuation of trabecular bone, thus enabling visualization of subtle changes in bone marrow. BME images can be used to identify occult fractures. Visualization of BME can be used to identify fractures or bone focal lesions [19]. 

#### 2.2.2. Energy-Selective Images

Polychromatic X-ray beams are composed of photons at many energy levels that form the X-ray spectrum. Due to this material-specific information of DECT, the density of each voxel can be extrapolated to a certain energy level to generate virtual monochromatic (VMI) series. These can simulate how CT images would look if a monochromatic beam of X-rays at a chosen single energy was used for imaging. This type of image may improve image quality, increasing the contrast–noise ratio and reducing artifacts (such as beam hardening at higher keV values). When using lower energy levels, the energy level can be shifted close to the k-edge of iodine (36 keV), which may improve the visualization of contrast-enhanced lesions and reduce the iodine contrast dose administered. This feature can be useful in patients with impaired renal function [1,2,3,4,5,6,7,8] (Table 2).

DECT imaging also allows calculation of mass density (rho) and Zeff information. The Zeff image measures the average atomic number of a tissue, which is fundamental in radiotherapy planning [15]. Furthermore, preliminary results suggest that atomic numbers could be useful to discriminate non-enhancing from enhancing renal masses on effective atomic number maps. Mileto et al. [23] concluded that the value of 8.36 was the optimal threshold, with the enhancing masses showing higher values.

#### 2.2.3. Polychromatic-Like Images

In addition to the material- and energy-specific images, a mixture of low- and high-energy acquisitions is used to generate a single set of blended images similar to conventional single-energy CT images (i.e., 120 kVp-like images) to be used for routine diagnosis. They simulate the standard 120 kVp dataset in polychromatic CT combining high-contrast from low keV images with lower noise in higher keV images [1,2,3,4,5,6,7,8,9].

### 2.3. Technical Solutions for Acquiring DECT Imaging

The advance in technology has allowed for the development of different CT scanners in the field of DECT. Currently, all major vendors offer CT systems capable of DECT acquisition. However, commercially available DECT platforms have hardware and software differences that may influence the spectral performance and, consequently, lesion quantification and characterization [4,6,7].

The currently available advanced DECT systems fall into two main categories: source-based or detector-based:

- Source-based DECT systems:
  - Dual-source DECT with two X-ray tubes/detectors arranged perpendicular to each other.
  - DECT with rapid tube voltage switching alternating between high and low energies multiple times within the same rotation.
- Detector-based DECT systems:
  - Dual-layer DECT with a single X-ray tube and two layers of detectors, with a top layer detecting the low-energy photons and the bottom layer detecting high-energy photons.

**Figure 6:**
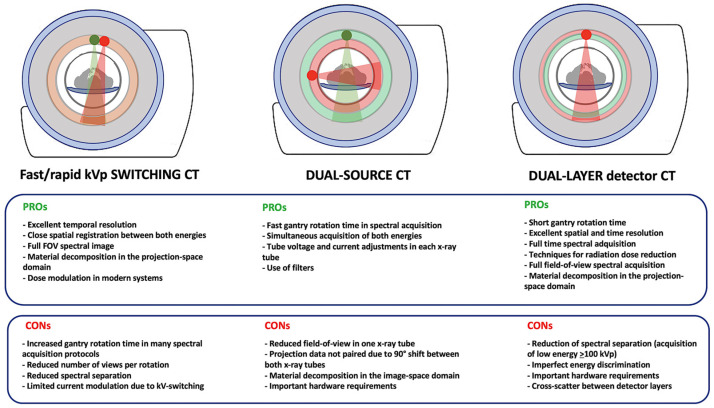
Differences between the main available advanced DECT systems and advantages (PROs) and disadvantages (CONs) of each.

The differences in hardware and software configurations across various DECT systems influence technical features, such as temporal resolution, coverage, and dose reduction, and impact spectral separation, material decomposition and quantification, and the types of images that can be generated. However, there is no perfect system and each has its advantages and disadvantages in clinical practice that are summarized in Figure 6 [4,6,7,8,9,10].

## 3. Clinical Applications of DECT Imaging: Dos and Maybes

Dual-energy/spectral CT imaging can be considered as a CT-plus imaging technique and has evolved into a useful clinical tool. DECT offers numerous advantages over conventional CT, such as image optimization, artifact reduction, and the ability to provide additional information regarding tissue composition and enhancement. The multiple image types generated from DECT acquisition have opened a wide range of clinical applications that have already been established. In addition, many new advanced applications have emerged.

### 3.1. Dos: Current Clinical Applications of DECT

**Figure 7:**
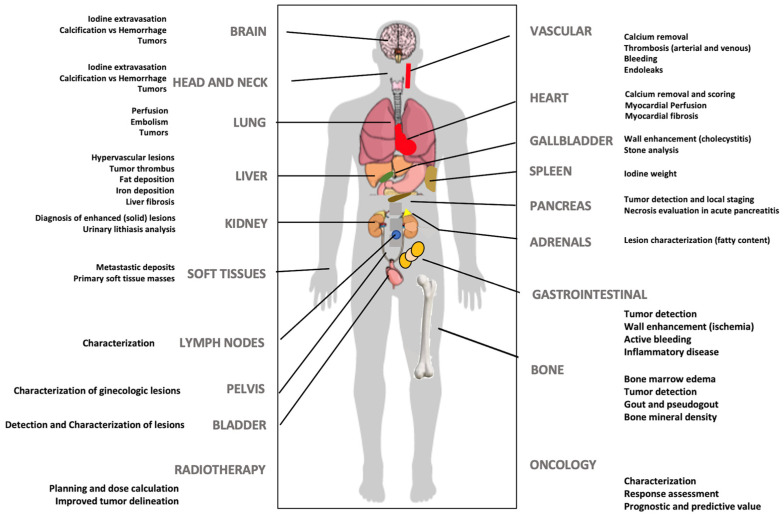
DECT imaging may offer clinical value in multiple clinical scenarios at different levels.

DECT offers the potential for improved lesion detection and characterization, superior determination of material composition, and more robust quantification. Furthermore, this technique enables a decrease in the amount of iodine contrast administered and a decrease in the radiation dose (eliminating the need for a non-contrast phase by generating VUE images), and can also reduce CT artifacts (e.g., beam hardening). Although a complete review of clinical applications of dual-energy imaging is outside the scope of this manuscript, DECT has demonstrated clear clinical benefits in many different anatomical areas and clinical scenarios, and offers a “*solving-problems*” tool allowing for an accurate characterization of incidental findings and obviating the need for further imaging, which may potentially reduce healthcare costs [1,2,3,4,5,6,7,8,9,10] (Figure 7).

### 3.2. Maybes: Advanced Applications of DECT

**Figure 8:**
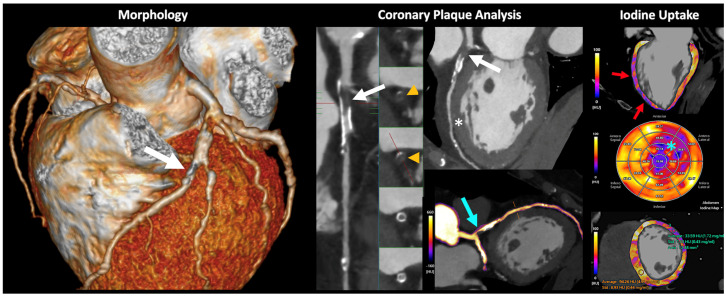
Volumetric cardiac DECT. “One-beat” spectral acquisition for coronary plaque evaluation. Cardiac CT morphologic (**left**), vascular (**middle**), and functional (**right**) analysis. Partially calcified plaque at the origin of the anterior descending coronary artery with intraplaque thrombosis (white arrows and orange arrowheads) generating severe stenosis (70–99%) with distal repermeabilization. The iodine map allows the depiction of the presence of iodine within the stenotic area (blue arrow). Myocardial iodine maps and polar maps, where a decrease in anterior and anterolateral midventricular, and anterior and lateral apical, uptake is identified (red arrows and blue asterisk, respectively). DECT also allows for iodine quantification and comparison to normal myocardium (bottom, right). This alteration is difficult to assess in conventional images (white asterisk).

DECT may offer a “one-stop-shop” imaging approach in various organs such as the heart and liver. In the case of cardiac imaging, dual-energy cardiac CT allows for a global cardiac assessment combining morphologic and functional analysis. DECT imaging offers high spatial resolution cardiac morphology; coronary plaque imaging and analysis with calcium scoring (including in post-contrast scans) and subtraction of calcium; improved evaluation of stent patency; calculation of fractional flow reserve; dynamic myocardial CT perfusion; assessment of myocardial extracellular space; and calculation of DECT-derived parameters such as iodine contrast uptake [24,25,26] (Figure 8). Furthermore, DECT improves image quality and can reduce both radiation and contrast media administration. The generation of iodine perfusion maps is clearly one of the most attractive contributions of cardiac DECT imaging. These maps can outline the iodine distribution within the myocardium, improving the evaluation of perfusion defects in infarct and ischemia. However, it is necessary to emphasize that iodine maps assess myocardial blood volume at a given time, and that they are not multiphase acquisitions such as dynamic perfusion CT [24,25,26].

**Figure 9:**
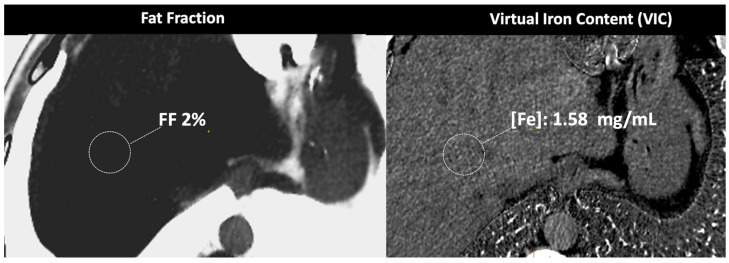
DECT-based assessment of fat and iron deposits. CT images demonstrate a fat fraction of 2% and a virtual iron concentration of 1.58 mg/mL, which rules out fatty infiltration or iron overload in this patient.

Another field of growing interest for the use of DECT is liver imaging. Advanced applications of liver DECT imaging include the assessment of fat and iron deposits and the calculation of the extracellular volume (ECV) (which is related to the degree of liver fibrosis). Although a non-contrast scan is preferred, recent studies have shown that DECT can accurately quantify liver fat even on contrast-enhanced images. In the case of liver iron content, both the attenuation difference of the liver between the low- and high-energy CT images or using an iron- or fat-specific material decomposition algorithm correlate well with the MR-based assessment of iron or fat accumulation [27,28,29,30] (Figure 9). The major limitations of the routine use of DECT in diffuse liver diseases are radiation and the additional software required for postprocessing. Finally, although DECT cannot directly detect or quantify fibrosis in the liver, the degree of hepatic fibrosis is strongly correlated with the ECV. The quantification of the ECV at a contrast-enhanced delayed phase (4–5 min) can be used to estimate the degree of hepatic fibrosis. A normalized iodine concentration of the liver (representing the ratio of the iodine concentration of the liver compared to that of the aorta) may reflect the amount of fibrosis based on the extent of iodine uptake [31].

**Figure 10:**
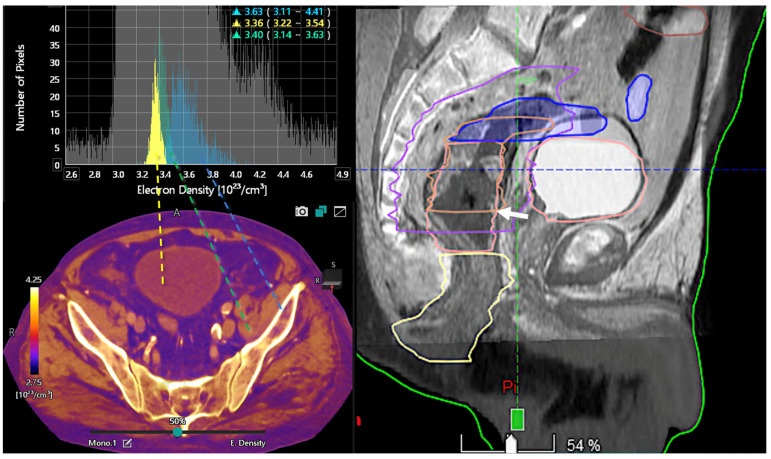
Color-coded electron density map based on DECT (**bottom**, **left**). Histogram analysis of electron density values at different locations (bladder lumen [water, yellow], muscle [green], and bone [blue]). Information on electron density is important for radiotherapy treatment planning in order to optimize the dose distribution and volume delineations. Sagittal fused CT and MR image (50% transparency) for therapy planning demonstrates the tumor (white arrow, **right** image).

Advanced applications of DECT may also provide quantitative measurements for radiotherapy planning, such as the Zeff and electron density of tissues for stopping power ratio calculation (SPR). DECT improves the accuracy of the SPR with an uncertainty of 1–2% in the proton range. DECT can be particularly useful for dose-delivery techniques with a steeper dose gradient, such as brachytherapy and proton therapy [32]. In particle therapy, SPR is significant for dose calculations because the planning target volume (PTV) margin includes the uncertainty determined based on the SPR. Protons release the maximum energy just before they stop penetration, making an accurate estimation of the SPR essential for therapy planning. Enhanced tumor visualization and delineation or artifact reduction using DECT have the potential to improve volume segmentation and dose calculation for radiation therapy planning in cancer treatment (Figure 10).

DECT technology has provided opportunities for new clinical applications, including the characterization of adrenal nodules, the detection of prostate and breast cancer, the assessment of lymph nodes (LNs), and the study of body composition.

**Figure 11:**
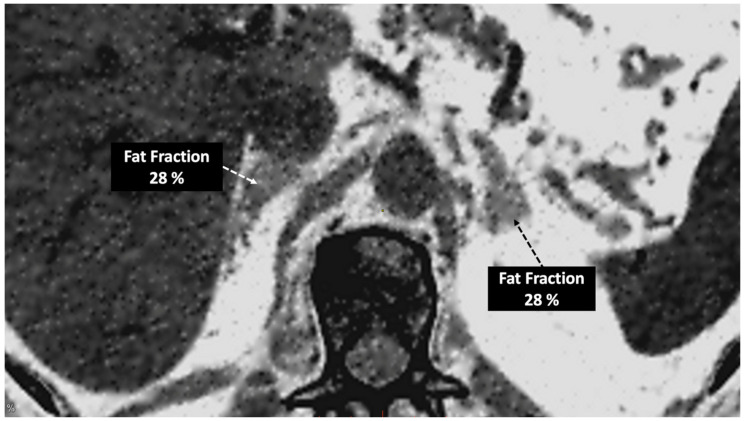
Adrenal adenoma. Fat fraction of adrenal lesions evaluated with DECT may be an alternative diagnostic tool to VUE attenuation using the traditional threshold of 10 HU or lower in the assessment of adrenal adenomas. Fat quantification DECT image evidences bilateral lipid-rich adrenal adenomas with increased fat fraction.

**Figure 12:**
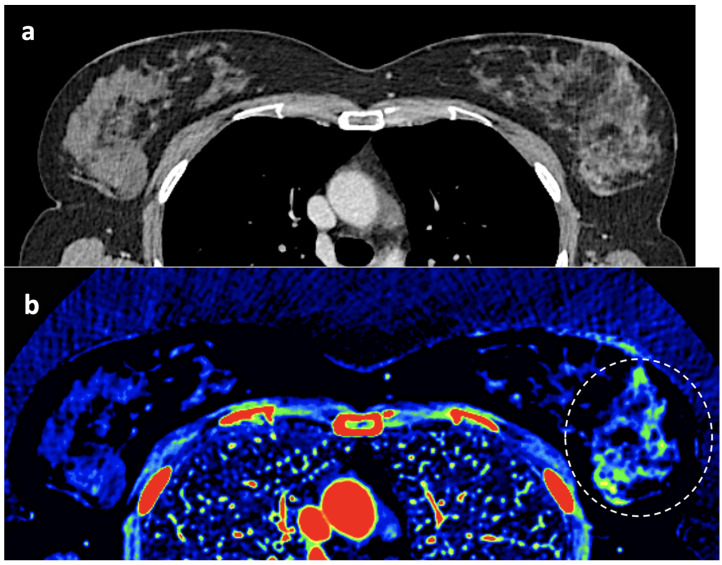
Breast 120 kVp-like (**a**) and color-coded iodine map (**b**) images. Breast lesion detection. Multicentric left breast invasive ductal carcinoma can easily be detected based on iodine uptake (dotted circle) compared to the 120 kVp-like image, where the diagnosis is challenging.

**Figure 13:**
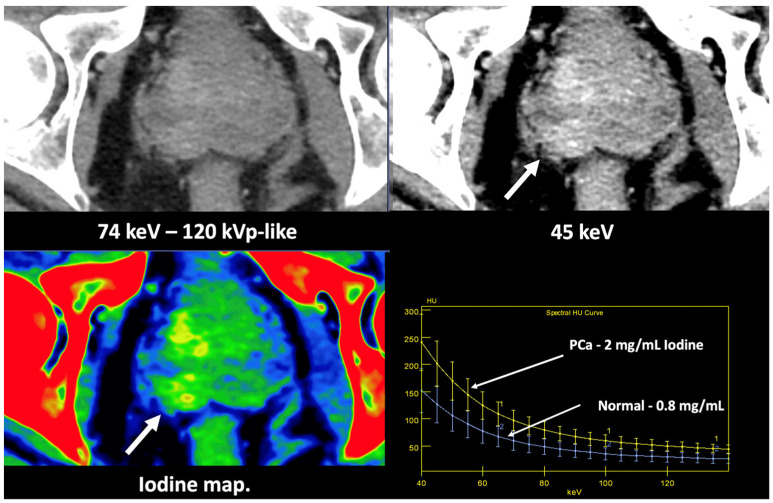
Prostate cancer (PCa) diagnosis. Incidental detection of a focal area of increased enhancement in the periphery of the prostate in contrast-enhanced CT may represent a clinically significant cancer (arrows) deserving of further workup. DECT imaging-based data such as a monoenergetic image at low energy values (e.g., 45 keV) and color-coded iodine map facilitate its detection. Note the difference between the spectral curve of the tumor (yellow curve, 1) and the normal parenchyma (blue curve, 2). Biopsy evidenced a prostate cancer having a Gleason score of 3 + 4.

**Figure 14:**
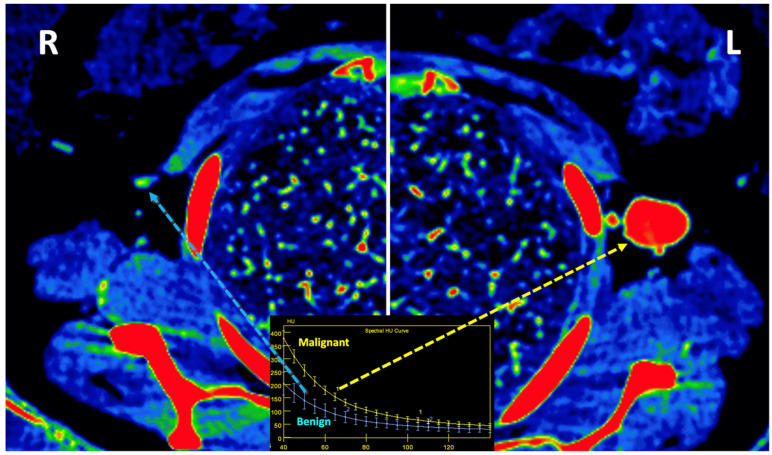
Lymph node (LN) imaging. Color-coded iodine maps of a left (L) axillary malignant LN (yellow arrow and yellow spectral curve, 1), which shows increased iodine uptake compared to a contralateral right (R) benign LN (blue arrow and blue spectral curve, 2).

**Figure 15:**
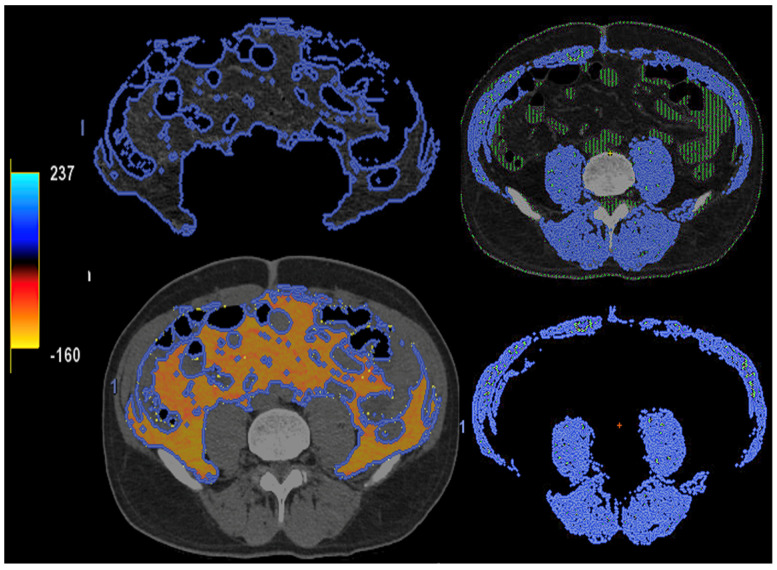
DECT-based segmentation of intraabdominal fatty tissue (orange areas) (**left** column) and segmentation of skeletal muscle volume (**right**, blue color).

**Figure 16:**
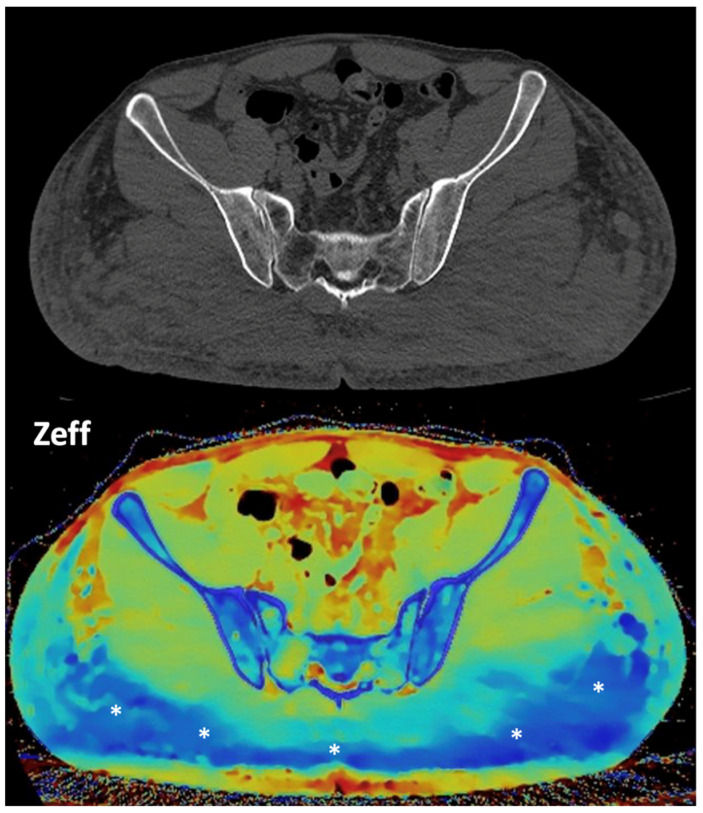
Gluteal silicone injection. Unenhanced CT image (**top**) shows a diffuse increase in density in both gluteal areas. Silicone deposition is better evaluated in a Zeff map (**bottom**), which separates silicone (blue areas, asterisks) from edema based on their different atomic numbers (silicon Z = 14).

- In the case of adrenal imaging, the fat fraction has higher sensitivity than VUE attenuation and the traditional threshold of 10 HU or lower for diagnosing adrenal adenomas. Loonis et al. [20] reported a fat fraction threshold of ≥23.8% with 100% specificity and 59% sensitivity (Figure 11). Furthermore, DECT-derived parameters can be used to differentiate adrenal adenoma from pheochromocytoma, or metastases based on the effect of lipid components on attenuation [33,34]. Finally, the iodine concentration can also be an imaging marker of dominant adrenal lesions in functional syndromes [35].
- Breast imaging. DECT seems to be a reliable tool for diagnosis and locoregional staging of breast cancer [36,37,38,39,40] (Figure 12). Klein et al. [37] found robust cut-off points for the differentiation of benign and malignant lesions (Zeff < 7.7, iodine content of <0.8 mg/mL). The DECT quantitative parameters may also be useful in predicting breast cancer invasiveness and histopathological and molecular subtypes of breast tumors. In the case of node staging, the similarity of quantitative DECT parameters between the primary lesion and axillary LNs may predict axillary metastasis in breast cancer [40,41].
- Currently, there is not a widely reported use of DECT in clinical management of prostate cancer. However, DECT imaging may facilitate the depiction of focal areas of increased enhancement in the periphery of the prostate in contrast-enhanced CT that may represent a clinically significant cancer and deserve further workup [42] (Figure 13).
- LN characterization is challenging in oncologic imaging. Apart from morphologic criteria, different DECT parameters have been used, including iodine concentration, fat fraction, and similarity to the primary tumor [41,43]. Sauter et al. [44] evaluated standard values for of iodine concentration for healthy LNs in different anatomic areas that could be used to differentiate between healthy and pathological LNs. Recent studies have suggested lower iodine concentration in metastatic LNs compared to benign LNs [45]. However, the value of DECT imaging in differentiating malignant from non-malignant LNs seems to be limited and depends on the tumor type and technical features such as the protocols used for acquisition and contrast injection (Figure 14).
- Imaging of body composition is another growing application of DECT imaging that can be used to improve the evaluation of muscle tissue, visceral adipose tissue (VAT), and subcutaneous adipose tissue (SAT) compartments. SAT and VAT assessment is of special interest in diseases related to metabolic syndrome and critically ill patients [46]. Moreover, sarcopenia is associated with a poorer prognosis in cancer patients [47]. Measuring fat fraction of the skeletal muscle by DECT is a new approach for the determination of muscle quality, an important parameter for the diagnostic confirmation of sarcopenia [48]. In the case of bone mineral density analysis, DECT can provide a more detailed analysis when compared with dual X-ray absorptiometry [49] (Figure 15). Finally, DECT can also be a useful tool for evaluating silicone implants (Figure 16). Silicone contains the heavier element silicon (Z value = 14), whereas soft tissue predominantly comprises lighter elements, depicting the presence of silicone within the soft tissues in cases of silicone gel breast implant rupture and LN silicone spread [50].

## 4. Limitations of DECT Imaging: Do Nots

DECT offers information about the presence of iodine uptake in tissues, providing valuable information for key clinical tasks such as the characterization of lesions, assessment of tumor vascularity and tissue perfusion, and monitoring of treatment response. However, despite the great advance in dual-energy imaging, the current DECT technology is still limited in many aspects.

First, although material decomposition algorithms can decompose unknown tissues into selected materials based on their attenuation plot at different energy levels, these assumptions may also explain many of the clinical limitations of DECT imaging. In the case of two-material decomposition algorithms, they assume that everything within the image is composed of different proportions of only two materials (e.g., iodine/water) and mathematically transform attenuation information into the concentrations of the two preselected materials that would be necessary to produce the measured attenuation level within each image voxel. It is important to consider that this should not be misinterpreted as an exact distribution of pure water and pure iodine; it simply means that their X-ray attenuation is the same as a specific combination of water and iodine. Algorithms for material decomposition (characterization) and quantification may work perfectly when the entire voxel is composed of only two materials, but human body composition is very heterogeneous, and a mixture of different tissues and materials is possible in the same voxel. In the clinical setting, human tissues usually contain multiple materials and we do not know, a priori, exactly what the number of materials in it is.

**Figure 17:**
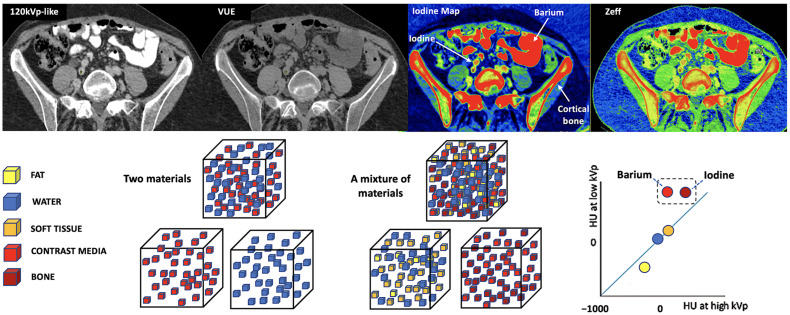
Difficult separation with DECT of materials of relatively close atomic numbers. When iodine and water are used as basis pairs of materials, elements such as barium or bone can be mistakenly classified as iodine. The VUE image suppresses iodine (Z = 53), but also subtracts barium (Z = 56) contrast and partially cortical bone. In the case of iodine concentration and Z-effective maps, they include all three elements. Algorithms for material decomposition and quantification work perfectly when only two materials are present (left bottom cubes and corresponding color coded circles, right bottom), but human body composition is heterogeneous (right bottom cubes). For example, calcium-containing voxels are seen on both material decomposition [iodine] and VUE DECT images, which affects the iodine quantification or subtraction.

Additionally, material decomposition algorithms based purely on the physics of the underlying attenuation process have several limitations [9,10,11]. If there is a sufficient difference in the Z- and K-edge of the two materials, DECT can be used to identify and differentiate them. However, it is challenging to separate materials having relatively close Z-values (e.g., iodine, barium, and bone) because they are going to show similar CT number ratios, which represent the ratio of the HU of the tissue or element acquired at low energy compared to that at high energy (Figure 17). Moreover, in two-material decomposition algorithms, the presence of another material can confound assessment. The attenuation of an additional material is assumed to be composed of different proportions of two materials within the basis pair selected, with the result, for example, that the attenuation of calcium is distributed roughly half and half between the iodine and water image contributions. This feature explains the visualization of calcium-containing voxels such as bone with iodine and water material-decomposition maps or in VUE images, or the limited value of iodine maps in areas of sclerotic bone. The increased “signal” of osseous or calcified structures depicted on iodine maps should not be misinterpreted as iodine. Generally, this pitfall can be easily avoided by cross-correlating the map to other maps (120 kVp-like, virtual unenhanced, or calcium maps).

Second, the main CT vendors have implemented markedly different hardware and software solutions for DECT imaging. Thus, there is a significant inter-vendor and inter-scanner variability in terms of algorithms for material labeling and quantification, so studies using different scanners should be interpreted with caution. There are two main methods of material decomposition: projection-based and image-based. Projection-based methods commonly provide better accuracy and image quality (artifact reduction). However, these methods need a perfect match of the projection datasets derived from both spectra (i.e., the same lines need to be measured at different energy levels), which is usually challenging. On the contrary, image-based methods are less quantitative but are easily applicable to the decomposition of three or more constituent materials. The variability in material-specific decomposition and quantification methods among manufacturers remains a limiting feature of dual-energy imaging standardization and comparison of results obtained with two different DECT systems. Thus, in the case of VUE attenuation values, inter-scan variation is higher in elements with high contrast enhancement such as vessels and kidneys [51].

Third, DECT-derived measurements are relative, not absolute, values. It is very important to consider that the different vendors’ solutions do not measure parameters in the same manner because the physical bases for image generation and analysis differ significantly between them. As a consequence, the iodine limit of detection varies across different scanners and vendors [16,52,53]. All DECT systems are able to depict iodine at concentrations between 0.3 and 0.5 mg/mL and to quantify at concentrations of 0.5–1.0 mg/mL, depending on the phantom size. However, it must be noted that the limit of iodine quantification (i.e., quantifying the amount of iodine present) is greater than the minimum iodine concentrations detectable (i.e., depicting the presence of iodine in a lesion), which limits the accuracy and the clinical value of iodine quantification. Perhaps the limit of detection can be used as a threshold of iodine concentration below which values may not represent true enhancement; however, it would be more clinically relevant to establish the iodine concentration above which there is definitive enhancement in a lesion [52,53]. These iodine concentration threshold values for iodine detection and quantification have been calculated based on phantom studies. Although CT phantoms are tissue-mimicking materials used to simulate and evaluate the interactions of ionizing radiation with human body tissues, it is very challenging to simulate the complexity of human tissues with iodine uptake. Therefore, thresholds published in the literature varied significantly between DECT systems. In the case of renal masses, published iodine content thresholds for renal mass enhancement vary between 0.5 mg/mL using a dual-source DECT system and 2.0 mg/mL with a rapid kV switching CT [54]. 

**Figure 18:**
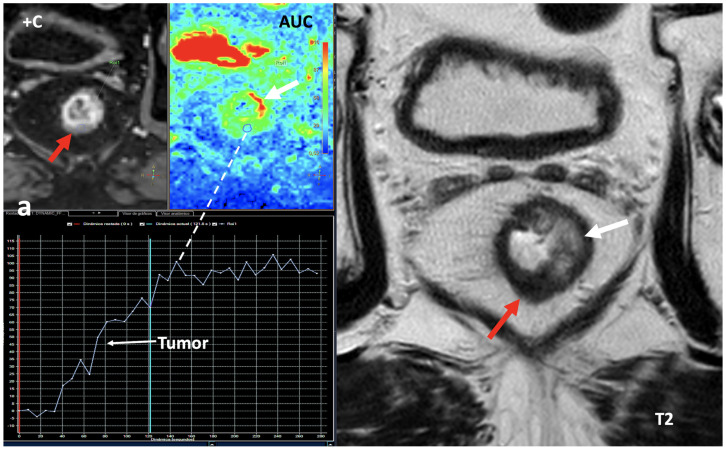

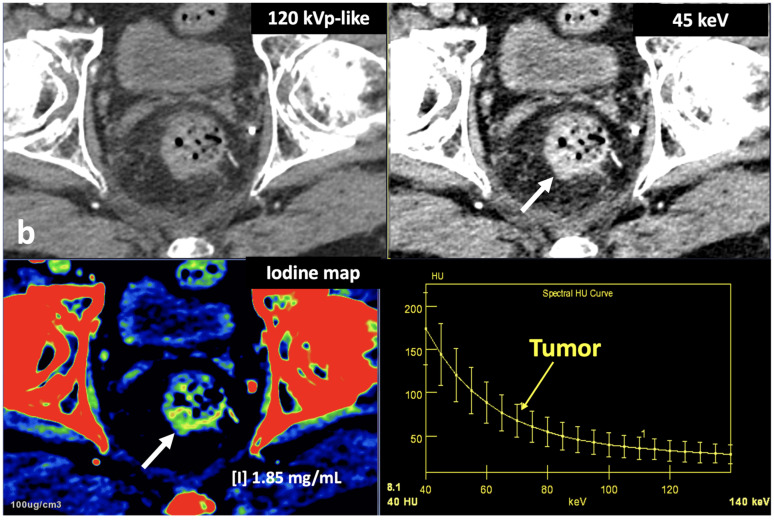
Rectal cancer following total neoadjuvant therapy. Screen capture of MRI sequences (**a**) including dynamic contrast-enhanced (+C) exam, area-under-the-curve (AUC) parametric map, time–intensity curve, and axial T2-weighted images demonstrates a residual lesion in the rectum (red arrow) with markedly hypointensity on T2 and with a type 1 curve of progressive enhancement suggesting fibrosis. Note secondary inflammatory changes (white arrows) with wall thickening on the left side of the rectum on T2 and increased signal on the AUC parametric map. DECT imaging (**b**) shows increased iodine uptake within the lesion (iodine concentration: 1.85 mg/mL). While DCE-MRI offers a dynamic assessment of tumor enhancement, iodine quantification is not a dynamic parameter, and only represents the amount of iodine contrast within an object in a concrete timing.

Fourth, the measurement of iodine concentration also depends on the contrast injection protocol, the time of acquisition, or factors from the patient themselves, such as cardiac input. The timing of image acquisition must be considered to avoid false-negative errors based on iodine quantification in slower and/or lesser enhancing lesions (i.e., papillary renal cancers). Iodine quantification is not a dynamic parameter, and only represents the amount of iodine contrast uptake within an object with a concrete timing following the administration of iodine contrast (Figure 18).

**Figure 19:**
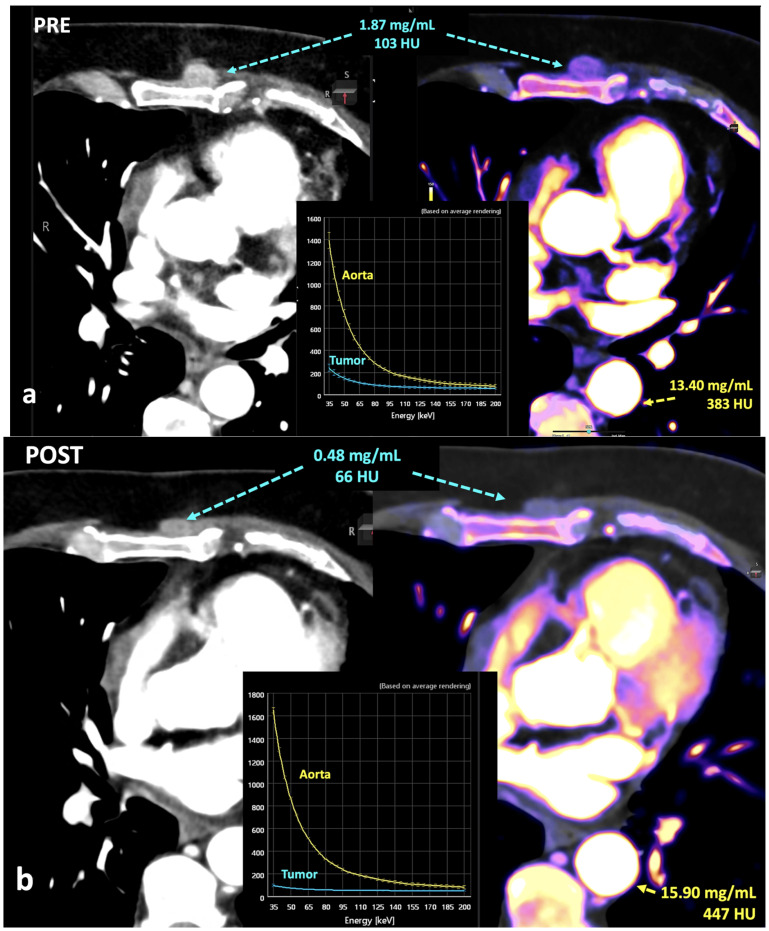
Breast cancer recurrence treated with hormotherapy and kinase inhibitors evaluated with DECT pre- (**a**) and post-therapy (**b**). A decrease in density and iodine concentration in the lesion is evident following therapy (**b**). Also note the different density and iodine concentration of the aorta in both studies, a difference in enhancement that in many cases might alter the degree of significance of the changes seen in the lesion.

In this setting, normalization to vessels could be a solution for a quantitative assessment of iodine concentration but the published data are discordant in this regard. Normalization can mitigate variability reducing physiological fluctuations in iodine distribution by using normalized iodine calculation, which represents the iodine concentration in an object divided by the iodine concentration in in a reference vessel such as the aorta [55,56] (Figure 19). Nevertheless, after normalization, the scanner type still has a significant effect on iodine variability in the pancreas and liver. Lennartz et al. [56] reported that iodine concentration also showed differences in variability between scanner types depending on the organ studied, with the least variability in the kidneys and highest variability in the liver [55,56].

**Figure 20:**
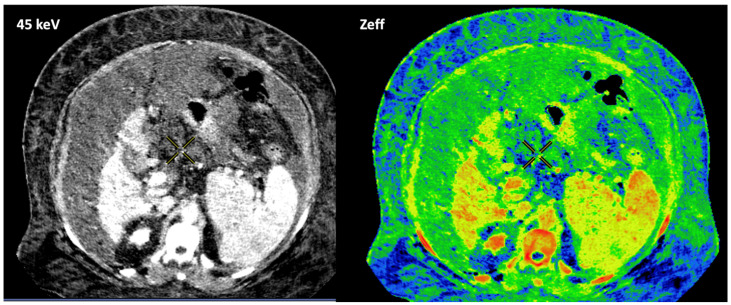
Patient’s body habitus and the presence of a massive ascite downgrades image quality and makes it impossible to adequately quantify the parameters (e.g., effective atomic number, Zeff) obtained.

Fifth, material-labeling and quantification accuracy is also influenced by other factors such as body habitus size (larger bodies may reduce the number of photons reaching the detectors and increase the image noise) and artifacts (Figure 20). Imaging artifacts that downgrade the quality of material decomposition and quantification and tissue characterization can originate from a wide range of different sources: technical features of CT acquisition (e.g., slower scanning speeds with some vendor’s DECT modes), image reconstruction algorithms, motion artifacts (which cause temporal misregistration of data from the two energies), or presence of metallic materials [10,57]. In the case of metallic implants, these devices preferentially absorb low-energy photons and leave a beam composed with higher-energy photons (beam hardening), which disrupts the calculation of the linear attenuation coefficient and causes streaking (dark bands) and cupping artifacts. Other important artifacts to consider are photon starvation and pseudo-enhancement. The former results from an insufficient number of low-energy photons reaching the detector, resulting in inadequate low-kilovoltage data for the characterization of materials. This artifact may appear in thick parts of the body where there may not be adequate X-ray penetration, such as the shoulders or pelvis, and areas of overlying metal. In the case of pseudo-enhancement, iodine content may erroneously be suggested in small non-enhancing lesions embedded in a background with increased iodine concentration due to a combination of beam hardening and partial voluming [10,57,58].

**Figure 21:**
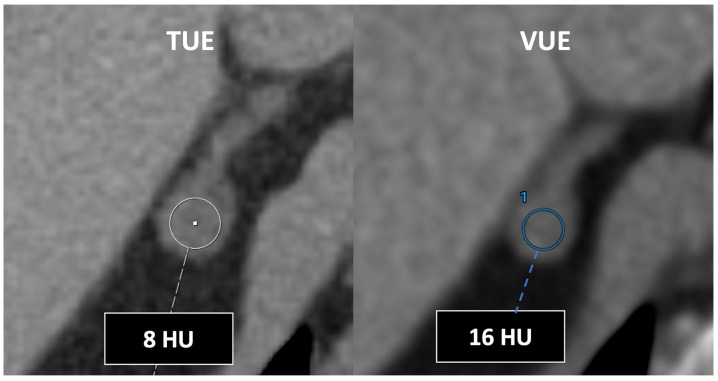
DECT-derived virtual unenhanced images may overestimate attenuation in adrenal nodules, resulting in low sensitivity for diagnosis of lipid-rich adenomas using the established 10 HU threshold. Note the different values obtained using true (TUE) (8 HU) and virtual (VUE) (16 HU) (blue circle, 1) unenhanced images.

Sixth, DECT enables computational removal of iodine content from CT images, generating VUE images. However, the algorithms used are not perfect. Attenuation measurements in VUE images may be higher than those calculated in true unenhanced acquisitions (TUEs) (especially in the case of fat with differences >10 UH) and non-reproductible among scanners (Figure 21). These differences between virtual unenhanced and true unenhanced attenuation values could be problematic in the case of adrenal lesions, limiting the adoption of the usual 10 HU threshold for characterization purposes [20,21]. Moreover, incomplete iodine removal in cases of very high iodine concentration may result in false positive findings suggesting malignancy [9,10,11]. Finally, iodine subtraction algorithms are prone to inadvertent subtraction of materials with a high Z-value like calcium. Calcifications and the size of calcifications tend to be underestimated in VUE images. This feature can reduce conspicuity of calcification in the pancreatic parenchyma in cases of chronic pancreatitis or in renal masses, affecting lesion interpretation.

**Figure 22:**
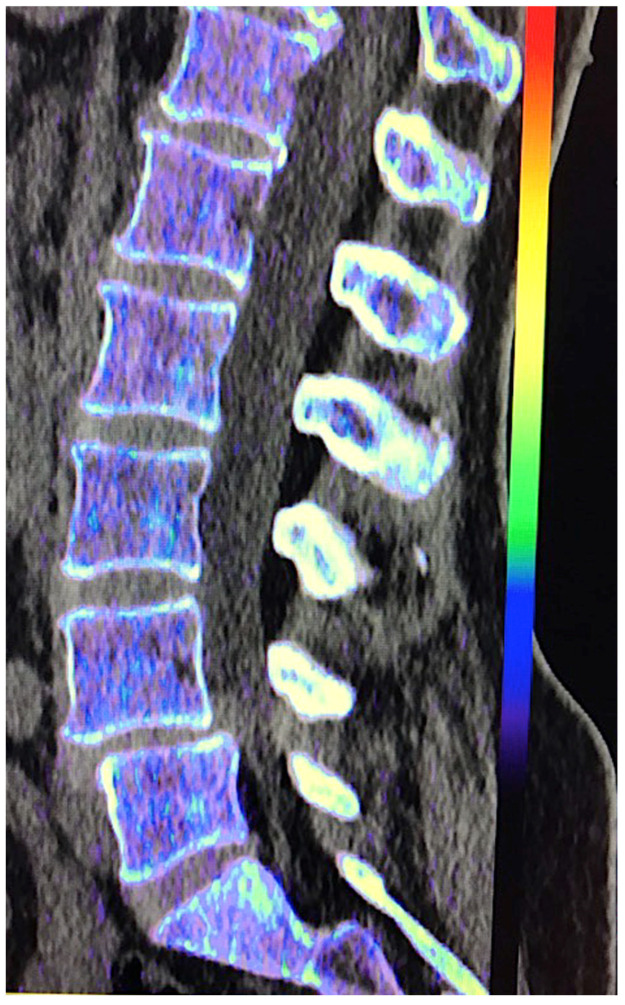
Virtual non-calcium (VNC) imaging allows the assessment of bone marrow. However, in cases of infiltrative tumors with low tumor burden, it may fail to depict bone marrow involvement. Color-coded VNC imaging of the lumbar spine was normal in a patient with a low level of infiltration by myeloma.

Finally, several additional materials, including calcium, fat, and uric acid, can be separated using DECT, but dual-energy findings are sometimes challenging to interpret as follows:

- VNCa improves CT sensitivity and specificity to assess bone marrow disorders. In VNCa imaging, the bone marrow attenuation mainly reflects the water and fat content on images. However, the optimal cutoff value for discrimination between infiltrated and normal bone marrow (ranging between −80 and 6 HU in the literature) and calcium suppression indices needs to be defined (Figure 22). VNCa imaging also shows limitations in evaluating bone marrow alterations in areas of sclerotic bone (e.g., close to the cortical bone) [22]. Apart of this, any bone marrow process (focal red marrow hyperplasia, malignant infiltrative lesions, etc.) that increases its attenuation can be misinterpreted as edema.
- DECT-derived fat fraction, a quantitative marker of fat content in the liver, correlates with histopathological examination, the reference standard for steatosis. Pathology assessment is based on the fraction of hepatocytes containing fatty vesicles: grade 0 (healthy, <5%), grade 1 (mild, 5–33%), grade 2 (moderate, 34–66%), and grade 3 (severe, >66%), while DECT evidences a substantially lower fatty liver content due to the simultaneous presence of fat, water, and soft tissue in the voxel. Pathologic data can be correlated with DECT-derived fat quantification and a conversion factor may aid in the prediction of the histopathological fat fraction based on fat quantification using DECT [30]. Patients with co-existing hepatic fat and iron overload represent a clinical challenge. In the presence of multiple material elements in the same voxel, it is still not clear whether the presence of fat and iron in the same voxel results in reduced performance of DECT [27].
- In the case of urates, monosodium urate foci may be either undetectable or underestimated by DECT with a low urate burden. This phenomenon has been reported in dense liquid tophi and calcified tophi due to subthreshold CT attenuation and obscuration of urate by calcium [59]. Concerning kidney lithiasis evaluation, inconsistent characterization may occur in tiny stones, as a result of the decreased signal from the stone which approaches the level of background noise. Furthermore, drainage device composition can also create stone mimics [18,60].

It is therefore necessary to take into consideration the aforementioned limitations in order to get the most out of the DECT technique in clinical practice. In addition, it is important to select the most appropriate pair of materials based on the clinical question to be answered.

## 5. The Future of DECT Imaging

Future DECT evolution will involve the improvement in dual-energy application workflow to extend the clinical value of DECT imaging. The main workflow concerns for DECT are the proliferation of image datasets and the need to use a thin-client server for image processing, which increase the time necessary to interpret a DECT scan. In this setting, DECT protocols need to be optimized to incorporate only the critical image series per protocol and minimize redundant datasets [61]. Finally, the development of cross-platform analysis methods may significantly decrease between-platform variability, allowing technical standardization [56,62]. All these advancements are set to streamline the routine DECT imaging process.

An evolving technology impacting nearly every aspect of dual-energy imaging is artificial intelligence (AI). AI will automate many critical aspects of the DECT process: acquisition (patient positioning, timing of acquisition, dose reduction), generation and modification of images (AI algorithms for reconstruction, denoising, and increasing resolution), and automation of time-consuming tasks for radiologists, such as image segmentation and quantitative analysis [63,64]. AI algorithms may improve image acquisition by performing data completion, which is accomplished by estimating missing projection data. Deep learning (DL) reconstruction may complete the views missing in the sinogram space and improves the image quality of sparse spectral-CT, a technique that can significantly reduce the radiation dose [65]. In the case of dual-source DECT scans, physical or hardware constraints limit the field of view covered by one of two X-ray tubes and cause lack of spectral information in the periphery of the patient. DL may extrapolate information outside the field of measurement of the second source–detector pair [66,67]. AI reduces image noise and improves DECT imaging quality [68,69,70,71]. Moreover, current material decomposition techniques suffer from excessive image noise and artifacts due to the dose limit in CT scanning and the noise magnification of the material decomposition process. DLR provides better noise containment for low keV images and AI techniques have been demonstrated that may improve material decomposition performance and detectability of low iodine concentrations [69,71,72]. Additionally, in clinical practice, AI has also shown to be useful in tumor detection, characterization, staging, and prognosis [70,73,74,75,76].

Finally, future applications of AI-based postprocessing techniques to DECT imaging include DECT-based thermometry and the generation of DECT parametric maps from conventional CT acquisitions. CT-based thermometry provides a non-invasive method for estimating temperature inside the human body by monitoring the attenuation value changes associated with temperature-dependent radiodensity. DL algorithms applied to DECT may improve this technique [77]. However, despite its clinical advantages, dual-energy imaging depends on expensive and not widely available CT systems. However, recent advancement in DL provides an enabling tool to generate DECT parametric maps using conventional CT scanners, which could greatly expand the usefulness of this technology [78,79]

Another important feature for DECT imaging will be the assessment of the relationships between DECT-derived parameters and biology. DECT provides qualitative and quantitative information about tissue composition and physiology. In the oncologic field, these parameters may play a pivotal role in tumor management, including diagnosis, characterization, grading, assessment of tumor invasiveness, staging, histopathological and molecular typing (ki-67 or PD-L1 expression, HER-2 status, K-RAS mutations, etc.), prognostic and predictive value, treatment planning, response assessment, and follow up [80,81,82,83,84,85,86,87,88,89,90,91]. In this setting, quantitative tumor biomarkers based on DECT include iodine concentration, Zeff values, and HU values [36,37,92,93,94,95].

- Data on physiological iodine uptake are still sparse. Physiologic iodine uptake values of organs and tissues may change depending on multiple features (body habitus, age, gender, etc.) that should be considered in the clinical use of DECT [37,95].
- Significant correlations were found between iodine concentration from DECT and perfusion CT-derived parameters such as blood volume and blood flow [92,93], although this correlation may vary at different acquisition times [94].
- Iodine concentration may be a surrogate marker of changes in tumor perfusion due to therapy [96]. Different iodine-related parameters have been proposed, such as concentration of intralesional iodine, vital iodine tumor burden, and (lesion volume × iodine concentration), which may be more sensitive than the evaluation criteria based on maximum diameter or change in CT value.
- Zeff is also a quantitative index for characterization of the composition of a voxel, although determining a biological correlation of these changes to tumor microenvironment is challenging.

**Figure 23:**
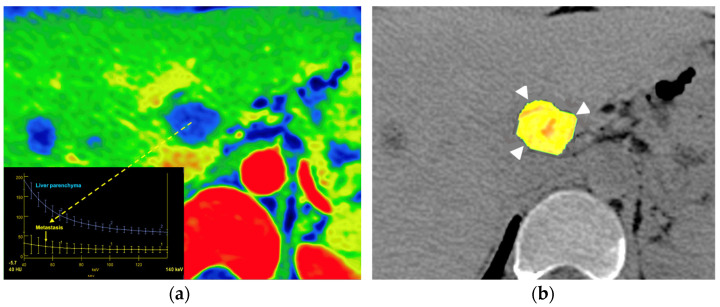
Metastatic colon cancer in the liver treated with chemotherapy. A color-coded iodine concentration map with a superimposed spectral curve (**a**) of the metastatic deposit (yellow arrow) compared to normal parenchyma shows no enhancement in the metastasis, suggesting extensive necrosis. A color-coded parametric map of entropy derived from texture analysis of iodine [no water] map superimposed on VUE image (**b**) evidence low entropy values in the metastatic deposit (postprocessing program *Olea Sphere*, *version 3.0*; *Olea Medical*, *La Ciotat*, *Francia*).

Moreover, computing advances have also facilitated the development of processes for high-throughput extraction of quantitative features that result in the conversion of images into mineable data and the subsequent analysis of voluminous CT image datasets for decision support. Radiomics and radiogenomics represent an innovative quantitative imaging approach that uses computer algorithms to extract and analyze a large number of quantitative features from radiological images [97]. Radiomics- and radiogenomics-based DECT analysis has been investigated for multiple applications in radiology, with a particular focus on oncologic imaging. Despite numerous published investigations and applications of radio(geno)mics analysis in the literature in various organ systems, there are currently little to no published data taking advantage of the rich quantitative datasets generated by DECT scans. The clinical value of DECT-derived radio(geno)mics features has been reported for diagnosis of malignancy, depicting genetic, molecular, and histological features in different tumor types; evaluating tumor invasiveness; predicting tumor staging; characterizing malignant LNs, predicting patient outcome and survival; and predicting and evaluating tumor response in different tumor types [98,99,100,101,102,103,104] (Figure 23).

**Figure 24:**
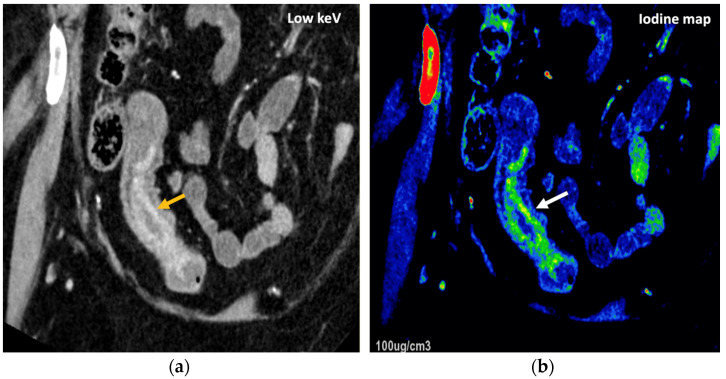
DECT offers definite advantages over single-energy CT to diagnose and assess disease severity of inflammatory bowel disease, particularly Crohn’s disease. A low-energy-level (45 keV) monochromatic reconstruction (**a**) and a color-coded iodine map (**b**) evidence increased enhancement and higher iodine concentrations, respectively, in the terminal ileum (orange and white arrows), which correlate with active inflammation in a patient with Crohn’s disease.

There are also preliminary data that support the possible value of DECT-derived features as imaging biomarkers in other non-oncologic applications, such as the diagnosis of pulmonary embolism, type 2 diabetes mellitus, osteoporosis, liver fibrosis, and carotid stenosis; the assessment of lung interstitial disease, Crohn’s disease activity, or acute pancreatitis; the characterization of adrenal masses; and the location and analysis of kidney stones [105,106,107,108,109,110,111,112,113] (Figure 24).

The robustness of features has been identified as pivotal for the clinical implementation. In this setting, the high repeatability of radiomics features has been established when keeping scan parameters and reconstruction conditions constant [114,115]. Unfortunately, most authors have evidenced that, although the repeatability of DECT radiomics features was high between scans–rescans, the inter-reproducibility of radiomics features between conventional CT and DECT, different types of images (VUE and monochromatic images), and DECT platforms was relatively low [114,115,116,117]. Apart from this, algorithms may influence radiomics reproducibility. Zhong et al. [115] evidenced that the use of deep learning image reconstruction algorithms may alter radiomics features’ reproducibility compared to conventional iterative reconstruction algorithms.

Finally, photon-counting CT (PCCT) represents the next stage in CT technology. PCCT is an emerging technique that uses photon-counting detectors to convert single incoming photons directly into an electrical pulse proportional to the photon’s energy. PCCT has the potential to overcome some of the limitations of DECT imaging, improving material decomposition, allowing the use of multiple contrast agents, filtering out the electronic noise of the image, and increasing spatial resolution. Currently, photon-counting technology offers clinical advantages in cardiovascular, thoracic, and musculoskeletal applications. Furthermore, the combination of ultra-high spatial resolution and spectral capabilities is expected to result in an improved performance of PCCT versus DECT for spectral separation and future applications of this technique to many anatomic regions and clinical scenarios [118,119].

## 6. Conclusions

The introduction of DECT in clinical routine has allowed some of the limitations of conventional CT to be overcome. In this setting, the added value of DECT has been widely validated in many clinical scenarios. DECT technology allows creation of numerous imaging datasets, including material- and energy-selective images. DECT imaging improves lesion detection and characterization, and facilitates superior determination of material composition and more robust quantification. However, a comprehensive grasp of the underlying basic principles of dual-energy imaging, its present technological constraints, and potential pitfalls (particularly artifacts) is imperative for an accurate interpretation of imaging findings. The potential of DECT imaging remains relatively untapped. Future technical developments might expand the full scope of clinical benefits offered by DECT, facilitating its seamless integration into clinical practice.

## Figures and Tables

**Table 1:** Material—selective images.

DECT Material Decomposition	Applications	Anatomical Region	Advantages
Iodine/Water or soft tissue	Virtual removal of iodinated contrast (virtual non enhanced, VUE) Dose reduction based on the avoidance of basal acquisitions Iodine quantification	General use throughout anatomy	Discrimination between enhancing and non-enhancing lesions Lesion characterization Response assessment Possible surrogate marker of perfusion parameters (iodine)
Iodine/Water or soft tissue/Fat	Fat quantification	Liver Kidney and adrenal Cardiovascular Full body composition Musculoskeletal	Fatty liver disease Fatty masses (kidney, adrenal, soft tissues) Vascular plaque characterization
Iron/Water or soft tissue/Fat	Iron quantification	Liver Musculoskeletal	Iron liver deposit Hemosiderin deposits (e.g., pigmented villonodular sinovitis)
Calcium/Water or soft tissue	Virtual non calcium—VNCa (bone/calcium removal) images Calcium quantification	Musculoskeletal Cardiovascular Abdominal Head and neck	Bone marrow edema Bone marrow lesions (e.g., myeloma) Vascular plaque evaluation Renal stones
Calcium/Hemorrhage	Hemorrhage evaluation	Head and neck	Brain hemorrhage vs. calcification
Uric acid/Calcium	Renal stone composition Gout	Abdominal imaging Musculoskeletal imaging	Differentiate calcific and uric acid-based renal stones Gout crystals deposit
Silicone/Soft tissue	Detection of silicone (silicon. Z value = 14)	Breast Soft tissue	Breast Implant Leaks Soft tissue implants

**Table 2:** Energy-selective imaging with DECT.

DECT Application	Applications	Anatomical Region	Advantages
Monoenergetic images	Simulate attenuation at a chosen single energy Improved image quality (increasing contrast, reducing artifacts) Reduction in dose of contrast material	General use throughout anatomy	- Reduction in proton-starving - and beam-hardening artifacts - Optimal image contrast/noise - Metal artifact reduction - Reduction in iodine load - Salvage of poor contrast studies - Improved detection and delineation of abnormalities
Effective atomic number (Zeff) and Electron density maps, (Rho-Z) maps	Material labeling (evaluation of effective atomic number (Zeff) and electron density (Rho) maps allow for the semiquantitative assessment of materials)	General use throughout anatomy	- Radiotherapy planning - Radiotherapy dose calculation - Stopping power ratio (protontherapy and brachitherapy) - Evaluation of cartilages and tendons - Characterization of renal masses (?)

## References

[1] Chung R., Dane B., Yeh B.M., Morgan D.E., Sahani D.V., Kambadakone A. (2023). Dual-Energy Computed Tomography: Technological Considerations. Radiol. Clin. N. Am..

[2] Forghani R., De Man B., Gupta R. (2017). Dual-Energy Computed Tomography: Physical Principles, Approaches to Scanning, Usage, and Implementation: Part 1. Neuroimaging Clin. N. Am..

[3] Forghani R., De Man B., Gupta R. (2017). Dual-Energy Computed Tomography: Physical Principles, Approaches to Scanning, Usage, and Implementation: Part 2. Neuroimaging Clin. N. Am..

[4] Borges A.P., Antunes C., Curvo-Semedo L. (2023). Pros and Cons of Dual-Energy CT Systems: “One Does Not Fit All”. Tomography.

[5] Agostini A., Borgheresi A., Mari A., Floridi C., Bruno F., Carotti M., Schicchi N., Barile A., Maggi S., Giovagnoni A. (2019). Dual-energy CT: Theoretical principles and clinical applications. Radiol. Med..

[6] Tatsugami F., Higaki T., Nakamura Y., Honda Y., Awai K. (2022). Dual-energy CT: Minimal essentials for radiologists. Jpn. J. Radiol..

[7] So A., Nicolaou S. (2021). Spectral Computed Tomography: Fundamental Principles and Recent Developments. Korean J. Radiol..

[8] Goo H.W., Goo J.M. (2017). Dual-Energy CT: New Horizon in Medical Imaging. Korean J. Radiol..

[9] Parakh A., Lennartz S., An C., Rajiah P., Yeh B.M., Simeone F.J., Sahani D.V., Kambadakone A.R. (2021). Dual-Energy CT Images: Pearls and Pitfalls. Radiographics.

[10] Parakh A., An C., Lennartz S., Rajiah P., Yeh B.M., Simeone F.J., Sahani D.V., Kambadakone A.R. (2021). Recognizing and Minimizing Artifacts at Dual-Energy CT. Radiographics.

[11] Patino M., Prochowski A., Agrawal M.D., Simeone F.J., Gupta R., Hahn P.F., Sahani D.V. (2016). Material Separation Using Dual-Energy CT: Current and Emerging Applications. Radiographics.

[12] Krauss B., Grant K.L., Schmidt B.T., Flohr T.G. (2015). The importance of spectral separation: An assessment of dual-energy spectral separation for quantitative ability and dose efficiency. Investig. Radiol..

[13] Sodickson A.D., Keraliya A., Czakowski B., Primak A., Wortman J., Uyeda J.W. (2021). Dual energy CT in clinical routine: How it works and how it adds value. Emerg. Radiol..

[14] Rajiah P., Parakh A., Kay F., Baruah D., Kambadakone A.R., Leng S. (2020). Update on Multienergy CT: Physics, Principles, and Applications. Radiographics.

[15] Jacobsen M.C., Thrower S.L., Ger R.B., Leng S., Court L.E., Brock K.K., Tamm E.P., Cressman E.N.K., Cody D.D., Layman R.R. (2020). Multi-energy computed tomography and material quantification: Current barriers and opportunities for advancement. Med. Phys..

[16] Jacobsen M.C., Cressman E.N.K., Tamm E.P., Baluya D.L., Duan X., Cody D.D., Schellingerhout D., Layman R.R. (2019). Dual-Energy CT: Lower Limits of Iodine Detection and Quantification. Radiology.

[17] Molwitz I., Leiderer M., Özden C., Yamamura J. (2020). Dual-Energy Computed Tomography for Fat Quantification in the Liver and Bone Marrow: A Literature Review. Rofo.

[18] Nourian A., Ghiraldi E., Friedlander J.I. (2020). Dual-Energy CT for Urinary Stone Evaluation. Curr. Urol. Rep..

[19] Gosangi B., Mandell J.C., Weaver M.J., Uyeda J.W., Smith S.E., Sodickson A.D., Khurana B. (2020). Bone Marrow Edema at Dual-Energy CT: A Game Changer in the Emergency Department. Radiographics.

[20] Loonis A.T., Yu H., Glazer D.I., Bay C.P., Sodickson A.D. (2023). Dual Energy-Derived Metrics for Differentiating Adrenal Adeno-mas From Nonadenomas on Single-Phase Contrast-Enhanced CT. AJR Am. J. Roentgenol..

[21] Ananthakrishnan L., Rajiah P., Ahn R., Rassouli N., Xi Y., Soesbe T.C., Lewis M.A., Lenkinski R.E., Leyendecker J.R., Abbara S. (2017). Spectral detector CT-derived virtual non-contrast images: Comparison of attenuation values with unenhanced CT. Abdom. Imaging.

[22] D’Angelo T., Albrecht M.H., Caudo D., Mazziotti S., Vogl T.J., Wichmann J.L., Martin S., Yel I., Ascenti G., Koch V. (2021). Virtual non-calcium dual-energy CT: Clinical applications. Eur. Radiol. Exp..

[23] Mileto A., Allen B.C., Pietryga J.A., Farjat A.E., Zarzour J.G., Bellini D., Ebner L., Morgan D.E. (2017). Characterization of Incidental Renal Mass With Dual-Energy CT: Diagnostic Accuracy of Effective Atomic Number Maps for Discriminating Nonenhancing Cysts From Enhancing Masses. AJR Am. J. Roentgenol..

[24] Danad I., Fayad Z.A., Willemink M.J., Min J.K. (2015). New Applications of Cardiac Computed Tomography: Dual-Energy, Spec-tral, and Molecular CT Imaging. JACC Cardiovasc. Imaging.

[25] Dell’Aversana S., Ascione R., De Giorgi M., De Lucia D.R., Cuocolo R., Boccalatte M., Sibilio G., Napolitano G., Muscogiuri G., Sironi S. (2022). Dual-Energy CT of the Heart: A Review. J. Imaging.

[26] De Santis D., Eid M., De Cecco C.N., Jacobs B.E., Albrecht M.H., Varga-Szemes A., Tesche C., Caruso D., Laghi A., Schoepf U.J. (2018). Dual-Energy Computed Tomography in Cardiothoracic Vascular Imaging. Radiol. Clin. N. Am..

[27] Marri U.K., Madhusudhan K.S. (2022). Dual-Energy Computed Tomography in Diffuse Liver Diseases. J. Gastrointest. Abdom. Radiol. ISGAR.

[28] Elbanna K.Y., Mansoori B., Mileto A., Rogalla P., Guimarães L.S. (2020). Dual-energy CT in diffuse liver disease: Is there a role?. Abdom. Radiol..

[29] Molwitz I., Campbell G.M., Yamamura J., Knopp T., Toedter K., Fischer R., Wang Z.J., Busch A., Ozga A.K., Zhang S. (2022). Fat Quantification in Dual-Layer Detector Spectral Computed Tomography: Experimental Development and First In-Patient Validation. Investig. Radiol..

[30] Xu J.J., Boesen M.R., Hansen S.L., Ulriksen P.S., Holm S., Lönn L., Hansen K.L. (2022). Assessment of Liver Fat: Dual-Energy CT versus Conventional CT with and without Contrast. Diagnostics.

[31] Marri U.K., Das P., Shalimar Kalaivani M., Srivastava D.N., Madhusudhan K.S. (2021). Noninvasive Staging of Liver Fibrosis Using 5-Minute Delayed Dual-Energy CT: Comparison with US Elastography and Correlation with Histologic Findings. Radiology.

[32] Kruis M.F. (2022). Improving radiation physics, tumor visualisation, and treatment quantification in radiotherapy with spectral or dual-energy CT. J. Appl. Clin. Med. Phys..

[33] Wang Y.L., Liu X.L., Liao Z.B., Lu X.M., Chen L.L., Lei Y., Zhang H.W., Lin F. (2023). Dual-energy spectral detector computed tomography differential diagnosis of adrenal adenoma and pheochromocytoma: Changes in the energy level curve, a phenomenon caused by lipid components?. Front. Endocrinol..

[34] Winkelmann M.T., Gassenmaier S., Walter S.S., Artzner C., Lades F., Faby S., Nikolaou K., Bongers M.N. (2022). Differentiation of adrenal adenomas from adrenal metastases in single-phased staging dual-energy CT and radiomics. Diagn. Interv. Radiol..

[35] Huang M., Yang D., Zhang Y., Zhang Y., Mu Y. (2023). The value of CT-based energy imaging to discriminate dominant side lesions in primary aldosteronism. Front. Endocrinol..

[36] Klein K., Schafigh D.G., Wallis G.M., Campbell M.G., Malter W., Schömig-Markiefka B., Maintz D., Hellmich M., Krug K.B. (2024). Assignment of the biological value of solid breast masses based on quantitative evaluations of spectral CT examinations using electron density mapping, Z effective mapping and iodine mapping. Eur. J. Radiol..

[37] Zopfs D., Graffe J., Reimer R.P., Schäfer S., Persigehl T., Maintz D., Borggrefe J., Haneder S., Lennartz S., Große Hokamp N. (2021). Quantitative distribution of iodinated contrast media in body computed tomography: Data from a large reference cohort. Eur. Radiol..

[38] Wang X., Liu D., Zeng X., Jiang S., Li L., Yu T., Zhang J. (2021). Dual-energy CT quantitative parameters for the differentiation of benign from malignant lesions and the prediction of histopathological and molecular subtypes in breast cancer. Quant. Imaging Med. Surg..

[39] Metin N.O., Balcı S., Metin Y., Taşçı F., Gözükara M.G. (2024). Correlation Between Quantitative Parameters Obtained by Dual Energy Spectral CT and Prognostic Histopathological Factors and Biomarkers in Breast Cancer. Clin. Breast Cancer.

[40] Volterrani L., Gentili F., Fausto A., Pelini V., Megha T., Sardanelli F., Mazzei M.A. (2020). Dual-Energy CT for Locoregional Staging of Breast Cancer: Preliminary Results. AJR Am. J. Roentgenol..

[41] Terada K., Kawashima H., Yoneda N., Toshima F., Hirata M., Kobayashi S., Gabata T. (2022). Predicting axillary lymph node metastasis in breast cancer using the similarity of quantitative dual-energy CT parameters between the primary lesion and axillary lymph node. Jpn. J. Radiol..

[42] Huang G., Lebovic G., Vlachou P.A. (2019). Diagnostic Value of CT in Detecting Peripheral Zone Prostate Cancer. AJR Am. J. Roentgenol..

[43] Yel I., D’Angelo T., Gruenewald L.D., Koch V., Golbach R., Mahmoudi S., Ascenti G., Blandino A., Vogl T.J., Booz C. (2024). Dual-Energy CT Material Decomposition: The Value in the Detection of Lymph Node Metastasis from Breast Cancer. Diagnostics.

[44] Sauter A.P., Ostmeier S., Nadjiri J., Deniffel D., Rummeny E.J., Pfeiffer D. (2020). Iodine concentration of healthy lymph nodes of neck, axilla, and groin in dual-energy computed tomography. Acta Radiol..

[45] Rizzo S., Radice D., Femia M., De Marco P., Origgi D., Preda L., Barberis M., Vigorito R., Mauri G., Mauro A. (2018). Metastatic and non-metastatic lymph nodes: Quantification and different distribution of iodine uptake assessed by dual-energy CT. Eur. Radiol..

[46] Tolonen A., Pakarinen T., Sassi A., Kyttä J., Cancino W., Rinta-Kiikka I., Pertuz S., Arponen O. (2021). Methodology, clinical applications, and future directions of body composition analysis using computed tomography (CT) images: A review. Eur. J. Radiol..

[47] Chianca V., Albano D., Messina C., Gitto S., Ruffo G., Guarino S., Del Grande F., Sconfienza L.M. (2022). Sarcopenia: Imaging assessment and clinical application. Abdom. Radiol..

[48] Molwitz I., Leiderer M., McDonough R., Fischer R., Ozga A.K., Ozden C., Tahir E., Koehler D., Adam G., Yamamura J. (2021). Skeletal muscle fat quantification by dual-energy computed tomography in comparison with 3T MR imaging. Eur. Radiol..

[49] Mallinson P.I., Coupal T.M., McLaughlin P.D., Nicolaou S., Munk P.L., Ouellette H.A. (2016). Dual-Energy CT for the Musculoskeletal System. Radiology.

[50] Glazebrook K.N., Doerge S., Leng S., Drees T.A., Hunt K.N., Zingula S.N., Pruthi S., Geske S., Carter R.E., McCollough C.H. (2019). Ability of Dual-Energy CT to Detect Silicone Gel Breast Implant Rupture and Nodal Silicone Spread. AJR Am. J. Roentgenol..

[51] Lennartz S., Parakh A., Cao J., Kambadakone A. (2021). Longitudinal reproducibility of attenuation measurements on virtual un-enhanced images: Multivendor dual-energy CT evaluation. Eur. Radiol..

[52] Taylor R.E., Mager P., Yu N.C., Katz D.P., Brady J.R., Gupta N. (2019). Iodine quantification and detectability thresholds among major dual-energy CT platforms. Br. J. Radiol..

[53] Hindman N.M. (2019). How Low Can We Go? The Very Low Limits of Iodine Detection and Quantification in Dual-Energy CT. Radiology.

[54] Morgan D.E. (2018). The Role of Dual-Energy Computed Tomography in Assessment of Abdominal Oncology and Beyond. Radiol. Clin. N. Am..

[55] Patel B.N., Vernuccio F., Meyer M., Godwin B., Rosenberg M., Rudnick N., Harring S., Nelson R., Ramirez-Giraldo J.C., Farjat A. (2019). Dual-Energy CT Material Density Iodine Quantification for Distinguishing Vascular From Nonvascular Renal Lesions: Normalization Reduces Intermanufacturer Threshold Variability. AJR Am. J. Roentgenol..

[56] Lennartz S., Cao J., Pisuchpen N., Srinivas-Rao S., Locascio J.J., Parakh A., Hahn P.F., Mileto A., Sahani D., Kambadakone A. (2024). Intra-patient variability of iodine quantification across different dual-energy CT platforms: Assessment of normalization techniques. Eur. Radiol..

[57] Coupal T.M., Mallinson P.I., Gershony S.L., McLaughlin P.D., Munk P.L., Nicolaou S., Ouellette H.A. (2016). Getting the Most From Your Dual-Energy Scanner: Recognizing, Reducing, and Eliminating Artifacts. AJR Am. J. Roentgenol..

[58] Soesbe T.C., Ananthakrishnan L., Lewis M.A., Duan X., Nasr K., Xi Y., Abbara S., Leyendecker J.R., Lenkinski R.E. (2018). Pseudoenhancement effects on iodine quantification from dual-energy spectral CT systems: A multi-vendor phantom study regarding renal lesion characterization. Eur. J. Radiol..

[59] Ahn S.J., Zhang D., Levine B.D., Dalbeth N., Pool B., Ranganath V.K., Benhaim P., Nelson S.D., Hsieh S.S., FitzGerald J.D. (2021). Limitations of dual-energy CT in the detection of monosodium urate deposition in dense liquid tophi and calcified tophi. Skelet. Radiol..

[60] Jepperson M.A., Cernigliaro J.G., Sella D., Ibrahim E., Thiel D.D., Leng S., Haley  W.E. (2013). Dual-energy CT for the evaluation of urinary calculi: Image interpretation, pitfalls and stone mimics. Clin. Radiol..

[61] Megibow A.J., Kambadakone A., Ananthakrishnan L. (2018). Dual-Energy Computed Tomography: Image Acquisition, Processing, and Workflow. Radiol. Clin. N. Am..

[62] Cai L.M., Hippe D.S., Zamora D.A., Cao J., Parakh A., Kambadakone A.R., Xiao J.M., Wang S.S., Toia G.V., Gunn M.L. (2022). A Method for Reducing Variability Across Dual-Energy CT Manufacturers in Quantification of Low Iodine Content Levels. AJR Am. J. Roentgenol..

[63] McCollough C.H., Rajiah P.S. (2023). Milestones in CT: Past, Present, and Future. Radiology.

[64] Bousse A., Kandarpa V.S.S., Rit S., Perelli A., Li M., Wang G., Zhou J., Wang G. (2024). Systematic Review on Learning-based Spectral CT. IEEE Trans. Radiat. Plasma Med. Sci..

[65] Narita K., Nakamura Y., Higaki T., Kondo S., Honda Y., Kawashita I., Mitani H., Fukumoto W., Tani C., Chosa K. (2023). Iodine maps derived from sparse-view kV-switching dual-energy CT equipped with a deep learning reconstruction for diagnosis of hepatocellular carcinoma. Sci. Rep..

[66] Clark D.P., Schwartz F.R., Marin D., Ramirez-Giraldo J.C., Badea C.T. (2020). Deep learning based spectral extrapolation for dual-source, dual-energy x-ray computed tomography. Med. Phys..

[67] Maier J., Erath J., Sawall S., Fournié E., Stierstorfer K., Kachelrieß M. (2024). Raw data consistent deep learning-based field of view extension for dual-source dual-energy CT. Med. Phys..

[68] Li H., Li Z., Gao S., Hu J., Yang Z., Peng Y., Sun J. (2024). Performance evaluation of deep learning image reconstruction algorithm for dual-energy spectral CT imaging: A phantom study. J. X-ray Sci. Technol..

[69] Dabli D., Loisy M., Frandon J., de Oliveira F., Meerun A.M., Guiu B., Beregi J.-P., Greffier J. (2023). Comparison of image quality of two versions of deep-learning image reconstruction algorithm on a rapid kV-switching CT: A phantom study. Eur. Radiol. Exp..

[70] Chu B., Gan L., Shen Y., Song J., Liu L., Li J., Liu B. (2023). A Deep Learning Image Reconstruction Algorithm for Improving Image Quality and Hepatic Lesion Detectability in Abdominal Dual-Energy Computed Tomography: Preliminary Results. J. Digit. Imaging.

[71] Gong H., Baffour F.I., Glazebrook K.N., Tiegs-Heiden C.A., Rhodes N.G., Thorne J.E., Cook J.M., Kumar S., Fletcher J.G., McCollough C.H. (2022). Deep learning-based virtual noncalcium imaging in multiple myeloma using dual-energy CT. Med. Phys..

[72] Shi Z., Kong F., Cheng M., Cao H., Ouyang S., Cao Q. (2024). Multi-energy CT material decomposition using graph model improved CNN. Med. Biol. Eng. Comput..

[73] Shapira N., Fokuhl J., Schultheiß M., Beck S., Kopp F.K., Pfeiffer D., Dangelmaier J., Pahn G., Sauter A.P., Renger B. (2020). Liver lesion localisation and classification with convolutional neural networks: A comparison between conventional and spectral computed tomography. Biomed. Phys. Eng. Express.

[74] Li S., Yuan L., Lu T., Yang X., Ren W., Wang L., Zhao J., Deng J., Liu X., Xue C. (2023). Deep learning imaging reconstruction of reduced-dose 40 keV virtual monoenergetic imaging for early detection of colorectal cancer liver metastases. Eur. J. Radiol..

[75] Wang Y.W., Chen C.J., Huang H.C., Wang T.C., Chen H.M., Shih J.Y., Chen J.S., Huang Y.S., Chang Y.C., Chang R.F. (2021). Dual energy CT image prediction on primary tumor of lung cancer for nodal metastasis using deep learning. Comput. Med. Imaging Graph..

[76] Ge H.T., Chen J.W., Wang L.L., Zou T.X., Zheng B., Liu Y.F., Xue Y.J., Lin W.W. (2024). Preoperative prediction of lymphovascular and perineural invasion in gastric cancer using spectral computed tomography imaging and machine learning. World J. Gastroenterol..

[77] Heinrich A., Schenkl S., Buckreus D., Güttler F.V., Teichgräber U.K. (2022). Evaluation of the correlation between temperature and Hounsfield units (HU). CT-based thermometry with virtual monoenergetic images by dual-energy of fat, muscle and bone us-ing FBP, iterative and deep learning-based reconstruction. Eur. Radiol..

[78] Lyu T., Zhao W., Zhu Y., Wu Z., Zhang Y., Chen Y., Luo L., Li S., Xing L. (2021). Estimating dual-energy CT imaging from single-energ CT data with material decomposition convolutional neural network. Med. Image Anal..

[79] Kim S., Lee J., Kim J., Kim B., Choi C.H., Jung S. (2024). Conversion of single-energy CT to parametric maps of dual-energy CT using convolutional neural network. Br. J. Radiol..

[80] Foti G., Ascenti G., Agostini A., Longo C., Lombardo F., Inno A., Modena A., Gori S. (2024). Dual-Energy CT in Oncologic Imaging. Tomography.

[81] Ebrahimian S., Singh R., Netaji A., Madhusudhan K.S., Homayounieh F., Primak A., Lades F., Saini S., Kalra M.K., Sharma S. (2022). Characterization of Benign and Malignant Pancreatic Lesions with DECT Quantitative Metrics and Radiomics. Acad. Radiol..

[82] Liang G., Yu W., Liu S.Q., Xie M.G., Liu M. (2022). The value of radiomics based on dual-energy CT for differentiating benign from malignant solitary pulmonary nodules. BMC Med. Imaging.

[83] Barbara Krug K., Schömig-Markiefka B., Campbell G.M., Püsken M., Maintz D., Schlamann M., Klein K., Gabriel Schafigh D., Malter W., Hellmich M. (2022). Correlation of CT-data derived from multiparametric dual-layer CT-maps with immunohistochemical biomarkers in invasive breast carcinomas. Eur. J. Radiol..

[84] Azour L., Ko J.P., O’Donnell T., Patel N., Bhattacharji P., Moore W.H. (2022). Combined whole-lesion radiomic and iodine analysis for differentiation of pulmonary tumors. Sci. Rep..

[85] Jia Y., Xiao X., Sun Q., Jiang H. (2018). CT spectral parameters and serum tumour markers to differentiate histological types of cancer histology. Clin. Radiol..

[86] Manoharan D., Netaji A., Diwan K., Sharma S. (2020). Normalized Dual-Energy Iodine Ratio Best Differentiates Renal Cell Carcinoma Subtypes Among Quantitative Imaging Biomarkers From Perfusion CT and Dual-Energy CT. AJR Am. J. Roentgenol..

[87] Shi C., Yu Y., Yan J., Hu C. (2022). The added value of radiomics from dual-energy spectral CT derived iodine-based material de-composition images in predicting histological grade of gastric cancer. BMC Med. Imaging.

[88] Fan S., Li X., Zheng L., Hu D., Ren X., Ye Z. (2017). Correlations between the iodine concentrations from dual energy computed to-mography and molecular markers Ki-67 and HIF-1α in rectal cancer: A preliminary study. Eur. J. Radiol..

[89] Mahmoudi S., Koch V., Santos D.P.D., Ackermann J., Grünewald L.D., Weitkamp I., Yel I., Martin S.S., Albrecht M.H., Scholtz J.E. (2022). Imaging biomarkers to stratify lymph node metastases in abdominal CT—Is radiomics superior to dual-energy material decomposition?. Eur. J. Radiol. Open.

[90] Schramm N., Schlemmer M., Englhart E., Hittinger M., Becker C., Reiser M., Berger F. (2011). Dual energy CT for monitoring targeted therapies in patients with advanced gastrointestinal stromal tumor: Initial results. Curr. Pharm. Biotechnol..

[91] Drljevic-Nielsen A., Mains J.R., Thorup K., Andersen M.B., Rasmussen F., Donskov F. (2022). Early reduction in spectral dual-layer detector CT parameters as favorable imaging biomarkers in patients with metastatic renal cell carcinoma. Eur. Radiol..

[92] Kang H.J., Kim S.H., Bae J.S., Jeon S.K., Han J.K. (2018). Can quantitative iodine parameters on DECT replace perfusion CT parameters in colorectal cancers?. Eur. Radiol..

[93] Mulé S., Pigneur F., Quelever R., Tenenhaus R., Baranes L., Richard P., Tacher V., Herin E., Pasquier H., Ronot M. (2018). Can dual-energy CT replace perfusion CT for the functional evaluation of advanced hepatocellular carcinoma?. Eur. Radiol..

[94] Skornitzke S., Fritz F., Mayer P., Koell M., Hansen J., Pahn G., Hackert T., Kauczor H.U., Stiller W. (2018). Dual-energy CT iodine maps as an alternative quantitative imaging biomarker to abdominal CT perfusion: Determination of appropriate trigger delays for acquisition using bolus tracking. Br. J. Radiol..

[95] Yel I., Bucolo G.M., Mahmoudi S., Koch V., Gökduman  A., D′Angelo T., Grünewald L.D., Dimitrova M., Eichler K., Vogl T.J. (2024). Dual-Energy CT Iodine Uptake of Head and Neck: Definition of Reference Values in a Big Data Cohort. Diagnostics.

[96] Reginelli A., Del Canto M., Clemente A., Gragnano E., Cioce F., Urraro F., Martinelli E., Cappabianca S. (2023). The Role of Dual-Energy CT for the Assessment of Liver Metastasis Response to Treatment: Above the RECIST 1.1 Criteria. J. Clin. Med..

[97] Lafata K.J., Wang Y., Konkel B., Yin F.F., Bashir M.R. (2022). Radiomics: A primer on high-throughput image phenotyping. Abdom. Radiol..

[98] Lennartz S., Mager A., Große Hokamp N., Schäfer S., Zopfs D., Maintz D., Reinhardt H.C., Thomas R.K., Caldeira L., Persigehl T. (2021). Texture analysis of iodine maps and conventional images for k-nearest neighbor classification of benign and metastatic lung nodules. Cancer Imaging.

[99] Zheng Y., Han X., Jia X., Ding C., Zhang K., Li H., Cao X., Zhang X., Shi H. (2023). Dual-energy CT-based radiomics for predicting invasiveness of lung adenocarcinoma appearing as ground-glass nodules. Front. Oncol..

[100] Han D., Yu Y., He T., Yu N., Dang S., Wu H., Ren J., Duan X. (2021). Effect of radiomics from different virtual monochromatic images in dual-energy spectral CT on the WHO/ISUP classification of clear cell renal cell carcinoma. Clin. Radiol..

[101] Reinert C.P., Krieg E., Esser M., Nikolaou K., Bösmüller H., Horger M. (2021). Role of computed tomography texture analysis using dual-energy-based bone marrow imaging for multiple myeloma characterization: Comparison with histology and estab-lished serologic parameters. Eur. Radiol..

[102] Lenga L., Bernatz S., Martin S.S., Booz C., Solbach C., Mulert-Ernst R., Vogl T.J., Leithner D. (2021). Iodine Map Radiomics in Breast Cancer: Prediction of Metastatic Status. Cancers.

[103] Chen Y., Yuan F., Wang L., Li E., Xu Z., Wels M., Yao W., Zhang H. (2022). Evaluation of dual-energy CT derived radiomics signatures in predicting outcomes in patients with advanced gastric cancer after neoadjuvant chemotherapy. Eur. J. Surg. Oncol..

[104] Brendlin A.S., Peisen F., Almansour H., Afat S., Eigentler T., Amaral T., Faby S., Calvarons A.F., Nikolaou K., Othman A.E. (2021). A Machine learning model trained on dual-energy CT radiomics significantly improves immunotherapy response prediction for patients with stage IV melanoma. J. Immunother. Cancer.

[105] Foncubierta-Rodríguez A., Jiménez del Toro Ó.A., Platon A., Poletti P.A., Müller H., Depeursinge A. (2013). Benefits of texture analysis of dual energy CT for Computer-Aided pulmonary embolism detection. Annu. Int. Conf. IEEE Eng. Med. Biol. Soc..

[106] Jiang W., Pan X., Luo Q., Huang S., Liang Y., Zhong X., Zhang X., Deng W., Lv Y., Chen L. (2024). Radiomics analysis of pancreas based on dual-energy computed tomography for the detection of type 2 diabetes mellitus. Front. Med..

[107] Wang J., Zhou S., Chen S., He Y., Gao H., Yan L., Hu X., Li P., Shen H., Luo M. (2023). Prediction of osteoporosis using radiomics analysis derived from single source dual energy CT. BMC Musculoskelet. Disord..

[108] Ebrahimian S., Homayounieh F., Singh R., Primak A., Kalra M.K., Romero J.M. (2022). Spectral segmentation and radiomic features predict carotid stenosis and ipsilateral ischemic burden from DECT angiography. Diagn. Interv. Radiol..

[109] Choi B., Choi I.Y., Cha S.H., Yeom S.K., Chung H.H., Lee S.H., Cha J., Lee J.H. (2020). Feasibility of computed tomography texture analysis of hepatic fibrosis using dual-energy spectral detector computed tomography. Jpn. J. Radiol..

[110] Moon J.W., Bae J.P., Lee H.Y., Kim N., Chung M.P., Park H.Y., Chang Y., Seo J.B., Lee K.S. (2016). Perfusion- and pattern-based quantitative CT indexes using contrast-enhanced dual-energy computed tomography in diffuse interstitial lung disease: Relationships with physiologic impairment and prediction of prognosis. Eur. Radiol..

[111] Kim Y.S., Kim S.H., Ryu H.S., Han J.K. (2018). Iodine Quantification on Spectral Detector-Based Dual-Energy CT Enterography: Correlation with Crohn’s Disease Activity Index and External Validation. Korean J. Radiol..

[112] Mahmoudi S., Martin S., Koch V., Gruenewald L.D., Bernatz S., D'Angelo T., Vogl T.J., Booz C., Yel I. (2022). Value of Dual-Energy CT Perfusion Analysis in Patients with Acute Pancreatitis: Correlation and Discriminative Diagnostic Accuracy with Varying Disease Severity. Diagnostics.

[113] Huang J., Hou J., Yang W., Zhan M., Xie S., Li S., Li R., Wu S., He Y., Zhao W. (2023). Automatic Kidney Stone Composition Analysis Method Based on Dual-energy CT. Curr. Med. Imaging Rev..

[114] Euler A., Laqua F.C., Cester D., Lohaus N., Sartoretti T., Pinto Dos Santos D., Alkadhi H., Baessler B. (2021). Virtual Monoenergetic Images of Dual-Energy CT-Impact on Repeatability, Reproducibility, and Classification in Radiomics. Cancers.

[115] Zhong J., Pan Z., Chen Y., Wang L., Xia Y., Wang L., Li J., Lu W., Shi X., Feng J. (2023). Robustness of radiomics features of virtual unenhanced and virtual monoenergetic images in dual-energy CT among different imaging platforms and potential role of CT number variability. Insights Imaging.

[116] Lennartz S., O’Shea A., Parakh A., Persigehl T., Baessler B., Kambadakone A. (2022). Robustness of dual-energy CT-derived radiomic features across three different scanner types. Eur. Radiol..

[117] Chen Y., Zhong J., Wang L., Shi X., Lu W., Li J., Feng J., Xia Y., Chang R., Fan J. (2022). Robustness of CT radiomics features: Consistency within and between single-energy CT and dual-energy CT. Eur. Radiol..

[118] Douek P.C., Boccalini S., Oei E.H.G., Cormode D.P., Pourmorteza A., Boussel L., Si-Mohamed  S.A., Budde R.P.J. (2023). Clinical Applications of Photon-counting CT: A Review of Pioneer Studies and a Glimpse into the Future. Radiology.

[119] McCollough C.H., Rajendran K., Baffour F.E., Diehn F.I., Ferrero A., Glazebrook K.N., Horst K.K., Johnson T.F., Leng S., Mileto A. (2023). Clinical applications of photon counting detector CT. Eur. Radiol..

